# Flavokawain C Inhibits Cell Cycle and Promotes Apoptosis, Associated with Endoplasmic Reticulum Stress and Regulation of MAPKs and Akt Signaling Pathways in HCT 116 Human Colon Carcinoma Cells

**DOI:** 10.1371/journal.pone.0148775

**Published:** 2016-02-09

**Authors:** Chung-Weng Phang, Saiful Anuar Karsani, Gautam Sethi, Sri Nurestri Abd Malek

**Affiliations:** 1 Institute of Biological Sciences, Faculty of Science, University of Malaya, Kuala Lumpur, Malaysia; 2 Department of Pharmacology, Yong Loo Lin School of Medicine, National University of Singapore, 117600, Singapore, Singapore; University of Nebraska Medical Center, UNITED STATES

## Abstract

Flavokawain C (FKC) is a naturally occurring chalcone which can be found in Kava (*Piper methysticum* Forst) root. The present study evaluated the effect of FKC on the growth of various human cancer cell lines and the underlying associated mechanisms. FKC showed higher cytotoxic activity against HCT 116 cells in a time- and dose-dependent manner in comparison to other cell lines (MCF-7, HT-29, A549 and CaSki), with minimal toxicity on normal human colon cells. The apoptosis-inducing capability of FKC on HCT 116 cells was evidenced by cell shrinkage, chromatin condensation, DNA fragmentation and increased phosphatidylserine externalization. FKC was found to disrupt mitochondrial membrane potential, resulting in the release of Smac/DIABLO, AIF and cytochrome c into the cytoplasm. Our results also revealed that FKC induced intrinsic and extrinsic apoptosis via upregulation of the levels of pro-apoptotic proteins (Bak) and death receptors (DR5), while downregulation of the levels of anti-apoptotic proteins (XIAP, cIAP-1, c-Flip_L_, Bcl-xL and survivin), resulting in the activation of caspase-3, -8 and -9 and cleavage of poly(ADP-ribose) polymerase (PARP). FKC was also found to cause endoplasmic reticulum (ER) stress, as suggested by the elevation of GADD153 protein after FKC treatment. After the cells were exposed to FKC (60μM) over 18hrs, there was a substantial increase in the phosphorylation of ERK 1/2. The expression of phosphorylated Akt was also reduced. FKC also caused cell cycle arrest in the S phase in HCT 116 cells in a time- and dose-dependent manner and with accumulation of cells in the sub-G1 phase. This was accompanied by the downregulation of cyclin-dependent kinases (CDK2 and CDK4), consistent with the upregulation of CDK inhibitors (p21^Cip1^ and p27^Kip1^), and hypophosphorylation of Rb.

## Introduction

Colorectal cancer (CRC) is the third most common malignancy and fourth most common cause of cancer deaths worldwide, with an estimated 1.23 million new cases of CRC diagnosed and a mortality of 608000 in 2008. It is the third most common cancer in men and the second in women worldwide [1–2]. In Malaysia, CRC is the second most common cancer related mortality after breast cancer based on the Malaysia Cancer Statistics 2006 [3]. There are large geographic differences in the incidence of CRC globally. The highest mortality rates are in developed countries such as United States, Australia, Canada and Europe compared to developing countries [4]. However, the incidence of CRC is rapidly increasing in many Asian countries such as China, Japan, Korea and Singapore [2, 4–5].

**Fig 1:**
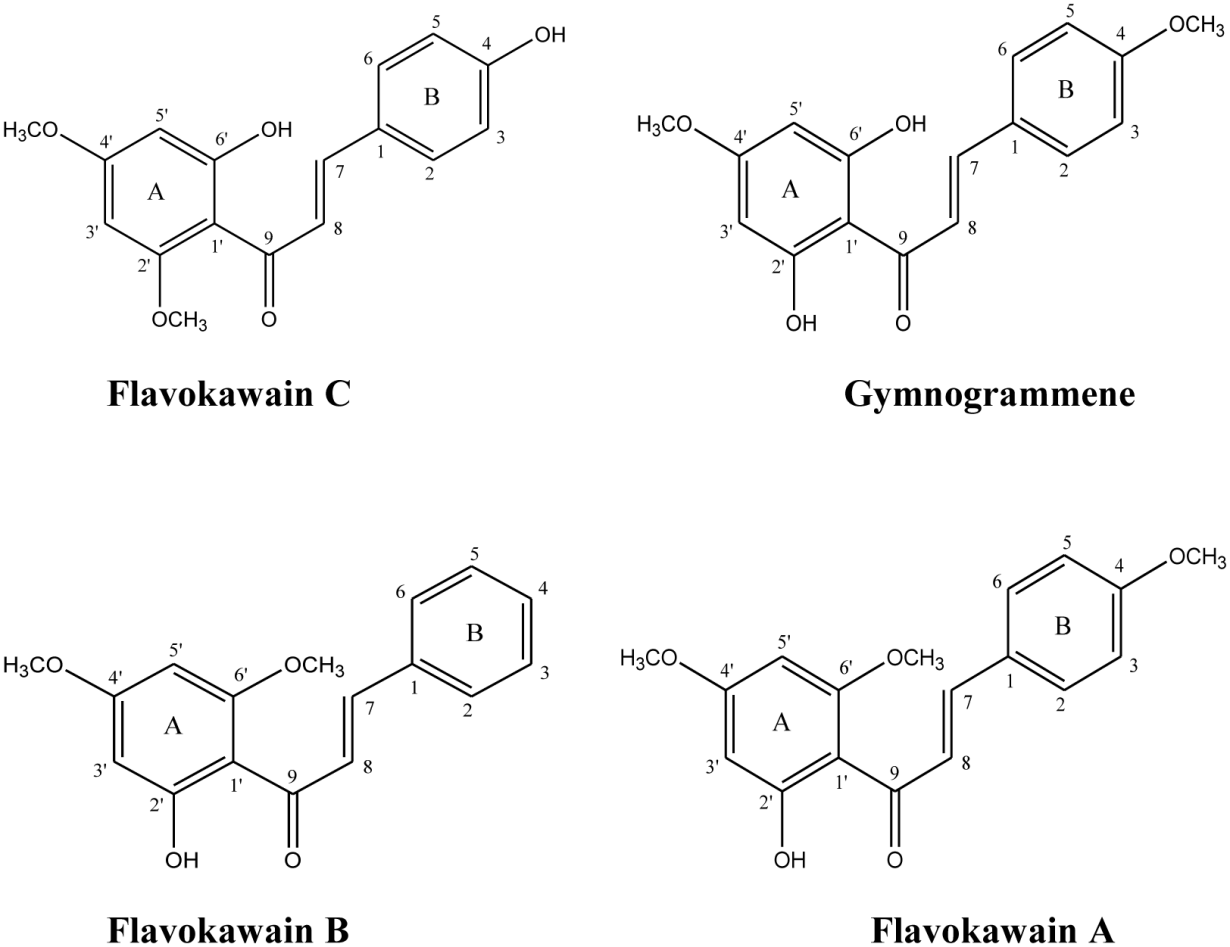
Chemical structure of flavokawain A, gymogrammene, flavokawain B, flavokawain C.

Chalcones have been shown to exhibit remarkable cytotoxic and apoptotic activities against a number of cancer cell lines. Among those reported were flavokawain A and B, xanthohumol and helichrysetin [6–8]. It was therefore of interest to investigate the anti-cancer potential of yet another chalcone, flavokawain C (FKC) and a structurally related chalcone, gymnogrammene (GMM). GMM only differs from FKC at C-2’ and C-4 in which the C-4 hydroxyl in FKC is replaced by a methoxy group whilst the C-2’ methoxyl group in FKC is replaced by a hydroxyl moiety (Fig 1).

FKC can be found in Kava (*Piper methysticum* Forst) root which grows naturally in Fiji and other South Pacific Islands where it constitute up to 0.012% of kava extracts [9]. In the Pacific Islands, Kava kava extracts have been traditionally prepared from macerated roots with water and coconut milk and used for centuries as a beverage for ceremonial purpose and social events without any side effects [10–11]. Kava-kava extracts have also been commercialized as a dietary supplement for treatment of stress, anxiety, insomnia, restlessness and muscle fatigue [12]. A previous study showed that FKC exhibited cytotoxic activity against three bladder cancer cell lines (T24, RT4 and EJ cells) with an IC_50_values of less than 17 μM [13]. Li *et al* reported that FKC showed mild cytotoxicity against human hepatoma cells (HepG2) and normal liver cells (L-02) with IC_50_ values of 57.04 and 59.08μM, respectively [14]. However, to the best of our knowledge, there has been no report on the apoptotic activities of FKC on any cancer or non-cancer cell lines.

Apoptosis or programmed cell death, is a mechanism by which cells are triggered to die to control cell proliferation in order to maintain normal cellular homeostasis or in response to DNA damage [15]. It is characterized by cell morphological changes such as cytoplasmic shrinkage, membrane blebbing, chromatin condensation, nuclear fragmentation followed by fragmentation into membrane-enclosed vesicles which are then engulfed by neighbouring cells or phagocytes, and biochemical changes such as externalization of phosphatidylserine, activation of caspases and breakdown of proteins [16–17]. There are three main apoptotic pathways: the intrinsic or mitochondrial pathway, the extrinsic or death receptor pathway and the endoplasmic reticulum stress pathway [18]. The intrinsic pathway involves a disruption of the mitochondria membrane potential triggered by intracellular stresses and also regulated by Bcl2 family proteins [19]. The extrinsic pathway involves binding of cell surface death receptors [(CD95 (Fas or Apo-1)/Trail/tumor necrosis factor (TNF) receptor 1 family proteins)] with their respective ligands [19]. These three pathways subsequently promote the activation of the caspase cascade which then trigger an ordered series of biochemical events that lead to cell changes and death [20]. Apart from that, inhibition of the signaling pathways that regulate the cell cycle progression may also lead to cytostatic and even apoptotic effects in cancer cells [21].

The mitogen-activated proteins kinases (MAPKs) and Akt signaling pathway play important roles in a variety of cellular processes including cell growth, differentiation, development, and apoptosis in response to extracellular stimuli and cellular stress [22]. Both Akt and MAPKs pathways are activated through a specific phosphorylation cascade. Akt (protein kinase/PKB) belongs to a family of serine/threonine kinases which mediate a downstream phosphoinositide 3-kinase (PI3K) signaling pathway and has become recognized as a potential molecular target for cancer therapy [23]. Activated Akt has been shown to promote tumor progression in human carcinoma including colorectal, lung, pancreas and breast through inhibition of apoptosis and promotion of cell cycle [23]. The MAPK subgroups, comprising of ERK1/2, JNK, and p38 MAPK, are known to relay, amplify and integrate signals from a diverse range of stimuli in controlling cellular proliferation, differentiation, development, inflammatory responses, apoptosis [24].

As such the present study was performed with the aim of characterizing the cytotoxic and apoptotic activities of FKC in HCT 116 colon carcinoma and its effects on the cell cycle *in vitro*. We show for the first time evidence that FKC induced substantial apoptosis and cell cycle arrest in colon carcinoma HCT 116 cell line and the underlying molecular mechanism involved.

## Materials and Methods

### Cell culture, compounds and reagents

Human colon carcinoma (HCT 116), human colon adenocarcinoma (HT-29), human lung carcinoma (A549), human cervical carcinoma (CaSki), human breast adenocarcinoma (MCF7) and non-cancerous human colon fibroblast (CCD-18Co) cell lines were purchased from the American Type Culture Collection (ATCC, USA). HCT 116 was cultured in McCoy’s 5A while HT-29, A549, CaSki and MCF7 were cultured in RPMI and all the medium were supplemented with 10% heat-inactivated fetal bovine serum (FBS; Sigma), 1% penicillin/streptomycin (100μg/ml; PAA Laboratories), 1% amphotericin B (250μg/ml; PAA Laboratories). CCD-18Co was cultured in Eagle minimum essential medium (MEM; Sigma) and supplemented with 10% heat-inactivated FBS, 1% Penicillin/Streptomycin (100μg/ml), 1% Amphotericin B (250μg/ml), 1% non-essential amino acid (100×; Sigma), and 1% sodium pyruvate (11mg/ml; Sigma). All the cells were maintained in a humidified incubator (5% CO_2_ in air at 37°C). FKC and GMM were obtained from the Extrasynthase (Genay, France) while cis-platin were obtained from Sigma and all compounds were dissolved in DMSO (Sigma).

### Antibodies and chemicals

The following antibodies were used: the primary antibodies for cleaved PARP-1 (cleaved p25), DR5, and Smac/DIABLO were purchased from GeneTex whereas AIF was purchased from Thermo Scientific. Antibodies against antibody against polyclonal caspase-3, monoclonal DR4, monoclonal p21, polyclonal p27, monoclonal Cdk2, polyclonal Cdk4, polyclonal cyclin D1, polyclonal cyclin E, polyclonal cIAP-1, polyclonal c-FLIPL, monoclonal survivin, polyclonal Bak, polyclonal Bax, monoclonal Bcl-xL, polyclonal Bid, polyclonal GADD153, monoclonal p-ERK, polyclonal ERK2 and polyclonal Akt1 were purchased from Santa Cruz. Antibodies against monoclonal caspase-8, monoclonal cytochrome c, monoclonal COX IV, polyclonal p-Rb, monoclonal Rb, polyclonal XIAP, monoclonal p-p38, polyclonal p38, polyclonal p-JNK (Thr183/Tyr185), monoclonal polyclonal JNK, p-Akt (Ser473) and β-actin were purchased from Cell Signaling Technology. The horseradish peroxidase (HRP)-labeled anti-mouse and anti-rabbit secondary antibodies were purchased from Santa Cruz. Bradford reagent was purchased from Bio-rad. Mitochondrial/cytosol fractionation kit was purchased from BioVision.

### Cyototoxic assay

Cells were seeded (4500 cells/well) in sterile 96-well plates in growth medium. They were incubated and cultured overnight to allow cell attachment. Following overnight incubation, the cells were treated with various concentrations of FKC or GM (5, 10, 21, 42, 84, 166 and 333μM) and further incubated for 24, 48 and 72 hours in 5% CO_2_ incubator. Untreated cells in 0.5% DMSO served as control. Cell viability was determined using the Sulphorhodamine B (SRB) assay, which is based on the measurement of the total protein mass of viable cells [25]. After the treatment period, the cells were fixed in 50 μl of ice-cold trichloroacetic acid (10% w/v) and incubated at 4°C for 1 hour. The cells were then washed and stained with 50μl of 0.4% SRB and left for 30 minutes at room temperature. They were then washed with 1% acetic acid (Merck) to remove any unbound dye and 100μl of 10mM Tris buffer (pH 10.5) was then added to dissolve protein-bound dye. The absorbance of dye eluted from viable cells was then measured (at 492 nm) using a microplate reader (BioTek). All experiments were performed in triplicates. Data were presented as means ± SD. Trypan blue exclusion assay was used to determine the numbers of live and dead cells in each treatment. The live and dead cells were counted in a 1:1 mixture of cell suspension and 0.4% (w/v) trypan blue solution in a hemocytometer chamber using a cell counter (Bio-Rad). IC_50_ was defined as the concentration (μM) of compound which caused 50% inhibition or cell death. A final concentration of 0.5% (v/v) DMSO was used (considered to be non-toxic). The IC_50_ value for each test sample was extrapolated from the graph of the percentage inhibition versus concentration of test sample. Dose- and time-dependent studies were performed to determine suitable doses and time for induction of apoptosis in cells. The percentage of inhibition and cell viability of each of the test samples was calculated according to the following formula:

$$\text{\% of inhibition = }\frac{{\text{OD}}_{\text{control}}{\text{ - OD}}_{\text{sample}}}{{\text{OD}}_{\text{control}}\;}\;\times 100\%$$

$$\text{\% cell viability = }\frac{{\text{ OD}}_{\text{sample}}}{{\text{ OD}}_{\text{control}}\;}\;\times 100\%$$

Where ODcontrol: Absorbance of negative control and ODsample: Absorbance of sample

### Morphological assessment of cell death by phase contrast and fluorescence microscopy

Cells (2.7 × 10^5^ cells/well) cultured in 6-well plates were treated with 0.5% DMSO or FKC at concentrations equivalent to; and also two and three times higher than the IC_50_ value for 48 hours. To evaluate the changes in cellular morphology, the cells were examined using a phase contrast inverted microscope (Zeiss AxioVert A1) after 48 hours at ×40 magnification. The morphological features of apoptotic cells observed included chromatin condensation, cell-volume shrinkage, and membrane-bound apoptotic bodies [26].

To evaluate changes in nuclear morphology induced by apoptosis, Hoechst 33342/propidium iodide (PI) double staining was used [27]. After 48 hours incubation, cells were harvested and washed with ice-cold PBS. The cells were then suspended in Hoechst 33342 (10μg/ml) and incubated at 37°C in a CO_2_ incubator for seven minutes. After incubation, the cells were counter-stained with propidium iodide (2.5μg/ml) and incubated in the dark for 15 minutes. The stained cells were then mounted onto glass microscope slides and observed immediately under fluorescence microscope (Leica DM160008). The images were captured with a digital camera (Leica DFC 310 FX). The cells were then classified as follows: (1) live cells (normal nuclei, blue chromatin with organized structure); early apoptotic cells (bright blue chromatin, which is highly condensed or fragmented); late apoptotic cells (bright pink chromatin, highly condensed or fragmented; necrotic (red, enlarged nuclei with normal structure) [28].

### Analysis of plasma membrane alteration

Apoptotic cells were quantified by Annexin V-FITC Apoptosis Detection Kit (BD Pharmingen) using flow cytometry. FITC-conjugated Annexin V was used to measure the loss of asymmetry of phosphatidylserine on apoptotic cell membranes while propidium iodide (PI) was used to differentiate early apoptotic from late apoptotic and necrotic cells [29]. Briefly, cells at a density of 1 x 10^6^ cells/well were treated with FKC in 0.5% DMSO at concentrations equivalent to; and also two and three times higher than the IC_50_values, for 24 and 48 hours. After treatment, adherent and floating cells were harvested and washed with PBS. The cells were then incubated in 100μl of annexin V-PI labeling solution containing 3μl of annexin V-FITC and 3μl of PI for 15 minutes at room temperature in the dark. After incubation, the cells were resuspended in 400μl of binding buffer before being analyzed using flow cytometer (Accuri C6) with cell counts of 10,000. The cell population were quantified in a percentage based on the four quadrants from a dot plot of FL1 (Annexin V) versus FL2 (PI): lower left (viable cells, Annexin V(-)/PI(-)), lower right (early apoptotic cells, Annexin V(+)/PI(-)), upper right (late apoptotic cells, Annexin V(+)/PI(+)) and upper left (dead cells/debris, PI(+), Annexin V(-)). The control was the well containing cells in 0.5% DMSO but without FKC.

### Analysis of changes in mitochondrial membrane potential (ΔΨ_m_)

The loss of mitochondrial membrane potential was assessed using lipophilic cationic fluorochrome JC-1 (5,5’,6,6’-tetrachloro-1,1’,3,3’-tetraethyl benzimidazolyl carbocyanine iodide). The assay was carried out using BD MitoScreen kit as per manufacturer’s protocol. Approximately 1 x 10^6^ cells were treated with FKC in 0.5% DMSO at concentrations equivalent to; and also two and three times higher than the IC_50_values for 24 and 48 hours. Following treatment, the cells were harvested and washed twice with PBS. The cells were then suspended in 500μl of JC-1 working solution and incubated at 37°C after which they were analyzed with flow cytometry (Accuri C6) where 10,000 events were recorded per analysis. In healthy cells, JC-1 accumulates as aggregates in the mitochondria and emits red fluorescence, whereas in apoptotic cells, the JC-1 remains in monomeric form in the cytoplasm and fluoresces green. The red and green fluorescence were detected at FL-2 and FL-1 channels, respectively in flow cytometer. The change in membrane potential was determined by calculating the ratio of mean fluorescence intensity between the FL1 and FL2 channels. The results were analyzed by calculating the ratio of JC-1 dimers to JC-1 monomers. A higher ratio indicated a higher membrane depolarization of mitochondria in cells. Untreated cells in 0.5% DMSO served as the control.

### Detection of DNA fragmentation by TUNEL assay

DNA fragmentation was assessed using the TUNEL (terminal deoxynucleotidyl transferase-mediated dUTP nick end-labelling) assay kit (APO-BrdU; invitrogen), according to the instructions provided by the manufacturer. Cells were grown in 60 mm petri dishes and exposed to FKC in 0.5% DMSO at concentrations equivalent to; and also two and three times higher than their IC_50_valuesfor 48 hours. Both detached and attached cells were harvested and fixed with 4% formaldehyde and permeabilized using 70% ethanol overnight. For detection of fragmented DNA, the cells were washed and incubated with DNA labeling solution containing TdT enzyme and Brd UTP for one hour at 37°C. After incubation, the cells were labeled with FITC-labeled anti-BrdU antibody followed by staining with propidium iodide/RNase for 30 minutes. The cells were then analyzed using Accuri C6 flow cytometer. Untreated cells in 0.5% DMSO served as the control.

### Assay for activation of Caspase-3/8/9

Caspases are key mediators of cell death. Caspase activity assay was performed using Caspases-3, -8 and -9 Staining Kit (CaspILLUME, Genetex). Briefly, cells at a density of 1×10^6^ were cultured in 60 mm petri dish. The cells were then treated with FKC in 0.5% DMSO at concentrations equivalent to; and also two and three times higher than their IC_50_ values for 48 hours. After incubation, the cells were washed and incubated with 1μl of *in situ* marker (FITC-DEVD-FMK for caspase-3, FITC-IETD-FMK for caspase-8 and FITC-LEHD-FMK for caspase-9) for 20 minutes in 5% CO_2_ at 37°C before being analyzed by flow cytometry (Accuri C6) and BD CFlow software. The results were analyzed by determining the percentage of activated caspase-3, -8 and -9 in comparison to the control. Untreated cells in 0.5% DMSO served as the control.

### Cell cycle analysis

Cell cycle arrest analysis was performed using PI staining and flow cytometry. This assay is based on the measurement of the DNA content of nuclei labeled with PI. Cells (2.7 ×10^5^ cells/well) were grown in a 6-well plate and exposed to FKC (20, 40 and 60μM). Both detached and attached cells were harvested and then pelleted by centrifugation. The cell pellets were fixed and permeabilized by suspension in 5 ml ice-cold 70% ethanol at -20°C overnight. Following incubation, the cells were washed twice with PBS and resuspended in 500 μl of staining buffer containing 50μg/ml propidium iodide, 100μg/ml RNase, 0.1% sodium citrate and 0.1% Trition-X-100) and incubated in the dark at room temperature for 30 minutes. Cell cycle phase distribution was determined using Accuri C6 flow cytometer (Accuri C6) and BD CFlow software. The DNA content of at least 15,000 cells was counted per sample and the percentage of cells in various phases (G_0_, G_1_, S and G_2_/M phases) of cell cycle was evaluated using Modfit software. Untreated cells in 0.5% DMSO served as the control.

### Mitochondrial/cytosolic isolation and proteins extraction

Isolation of cytosolic and mitochondrial proteins was performed according to the manufacturer’s instructions (BioVision). Briefly, the cells were seeded at 2 × 10^6^ cells per 25 mm^2^ culture flask treated with or without FKC (60μM) at 12, 24 and 48 hours. Cells were washed twice with ice-cold PBS and collected by centrifugation at 600×g for 5 minutes at 4°C. The cells were resuspended in cytosolic extraction buffer and incubated on ice for 10 minutes. After incubation, the cells were homogenized in an ice-cold dounce tissue grinder. Homogenates were centrifuged at 700×g for 10 minutes to remove unbroken cells. The supernatant was collected and centrifuged again at 10,000×g for 30 minutes at 4°C. The resulting supernatant was collected as cytosolic fraction. The pellet was then resuspended with mitochondrial extraction buffer and centrifuged at 10,000×g for 10 minutes and the supernatant was collected as the mitochondrial fraction. The protein concentrations of the fractions were measured using Bradford method (Bio-Rad Laboratories) and the fractionated protein were analyzed by western blotting.

### Western blot analysis

Western blot analysis was used to evaluate the levels of apoptosis related proteins in HCT 116 cells following the indicated FKC treatment. Cells seeded at 1×10^6^ cells per petri dish (60mm) treated with FKC (60μM) for 6, 12, 18, 24 and 48 hours after overnight incubation. Cells were washed with cold phosphate buffer saline (PBS) and harvested. Cells were lysed in lysis buffer containing 250mmol/L NaCl, 20mmol/L HEPES, 2mmol/L EDTA (pH 8.0), 0.5mmol/L EGTA, 0.1% Triton X-100, 1.5ug/mL aprotinin, 1.5ug/mL leupeptin, 1 mmol/L phenylmethylsulfonylfluoride (PMSF) and 1.5mmol/L NaVO4. Lysates were then centrifuged at 13,300 rpm, 4°C for 10 minutes and the supernatants were collected. Protein concentrations were measured with Bradford method (Bio-Rad Laboratories). Proteins were denatured by boiling for five minutes at 100°C. Equal amounts of protein (50μg) were loaded onto a 10% or 12% SDS-PAGE gel for electrophoresis and electroblotted onto a nitrocellulose membrane (Bio-Rad) and blocked with Blocking One (Nacalai Tesque, Inc). The membranes were probed with specific primary antibodies in a blocking buffer overnight at 4°C. After blocking, the blots were washed with Tris-buffered saline containing 0.1% Tween-20 (TBST) three times to remove unbound antibody, followed by incubation with HRP-conjugated secondary antibodies (1: 10,000 dilution) for 1 hour at room temperature. Protein bands were visualized using enhanced chemiluminescence (WesternBright ECL, Advansta) and images were captured on a ChemiDoc XRS Imaging System (Bio-rad Hercules, CA, USA). The membranes were stripped and reprobed with different antibodies as necessary. β-actin was used as the internal standard for the total cell lysate and cytoplasmic fractions, whereas COX IV was used as the control for the mitochondrial fractions. Densitometric quantification of the bands was performed using ImageJ software and the results were expressed as fold change relative to the control after normalization with β-actin.

### Statistical analysis

Data were expressed as mean ± SD of triplicates. Statistical analysis of data was performed using SPSS Statistics 17.0 and differences with a p<0.05 were considered significant. The following notion was used: * indicated p<0.05, compared with the non-treated group.

## Results

### FKC exerts cytotoxicity against human cancer cell lines and selectively inhibits the viability of HCT 116 cells

**Fig 2:**
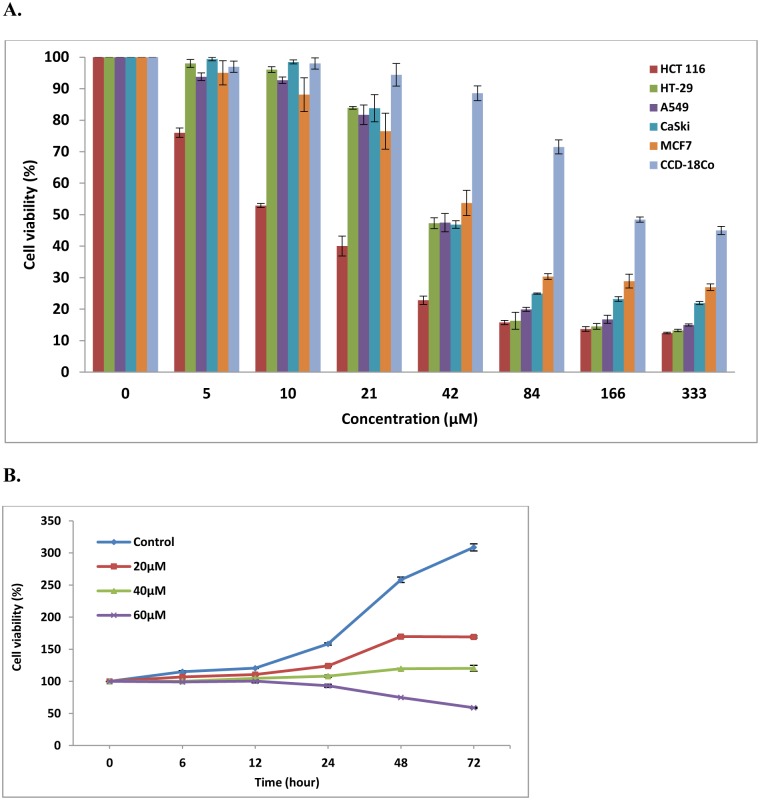
Inhibition of cell proliferation and viability by FKC in human cancer cell lines and a normal cell line. (A) Each cell lines were seeded at 4.5×10^3^ cells per well in 96-well plates. After 24 hours, the cells were treated with various concentration of FKC (5–333μM) for 72 hours, and the results were expressed as inhibition of proliferation which was determined by SRB assay as described in Materials and Methods. (B) HCT 116 cells (4.5×10^3^ cells/well) was seeded in a 96-well for 24 hours and then treated with FKC (20, 40 and 60μM) at increasing time points (6, 12, 24, 48 and 72 hours). All data shown are the mean±standard deviation (SD) of triplicates obtained from three independent experiments. Cell viability in FKC-treated cells was expressed as a percentage of viable cells compared to control cells.

We first investigated whether FKC can inhibit the growth of various human cancer cell lines (HCT 116, HT-29, A549, CaSki and MCF-7) and a normal colon cell line (CCD-18Co). The cells were exposed to various concentrations of FKC (5, 10, 21, 42, 84, 166, 333μM) for 72 hours and the cell viability was determined by SRB assay. IC_50_ value was defined as the concentration of drug that inhibited cell growth by 50%. As shown in Table 1 and Fig 2A, cell viability was reduced in the cancer cell lines tested in comparison with the control in a dose-dependent manner and HCT 116 cells was found to be most sensitive towards FKC (compared to other cancer cell lines) with an IC_50_value of 12.75±0.17μM. A comparison of the growth inhibitory effects of FKC against a chemotherapeutic drug, cisplatin is shown in Table 1. The IC_50_ of FKC was found to be comparable to cisplatin (IC_50_ 13.12±1.24μM) in HCT 116 cells. In the case of normal colon cells, FKC exhibited moderate cytotoxic effect against CCD-18Co cells which was less toxic compared to cisplatin, indicating a possible cytotoxic selectively towards colon cancer cells. In addition, a structurally related compound, gymnogrammene (GMM) was also evaluated for its cytotoxic activity against human cancer cell lines. GMM exhibited no cytotoxic activity against all other tested cell lines. Fig 2B shows that FKC decreased the growth of HCT 116 cells in a time-dependent manner at 20, 40 and 60μM. Growth was arrested after treatment with 60μM of FKC. The results show that FKC can suppress HCT 116 cells growth in a dose- and time-dependent manner. As such, HCT 116 cells were selected and subjected for further investigation on the potential underlying mechanism(s) of cell death induced by FKC.

**Table 1:** Cytotoxic activities of FKC and GMM on various cancer cell lines and human normal cell (CCD-18Co) for 72 hours treatment in comparison to Cisplatin.

Cell lines	IC_50_ in μM		
	FKC	GMM	Cisplatin
**HCT 116**	**12.75 ± 0.17**	**> 300**	**13.12 ± 1.24**
**HT-29**	**39.00 ± 0.37**	**> 300**	**34.35 ± 1.57**
**MCF-7**	**47.63 ± 5.93**	**> 300**	**> 300**
**A549**	**40.28 ± 2.11**	**> 300**	**19.47 ± 4.37**
**CaSki**	**39.88 ± 0.45**	**> 300**	**124.05 ± 3.08**
**CCD-18Co**	**160.86 ± 2.45**	**> 300**	**108.90 ± 2.91**

The IC_50_ value indicates a concentration of compounds which caused 50% reduction in cell viability based on SRB assay. Cisplatin was used as standard. Each value is expressed as mean±standard deviation of three replicates of three independent experiments.

### FKC causes cell death via induction of apoptosis in HCT 116 cells

**Fig 3:**
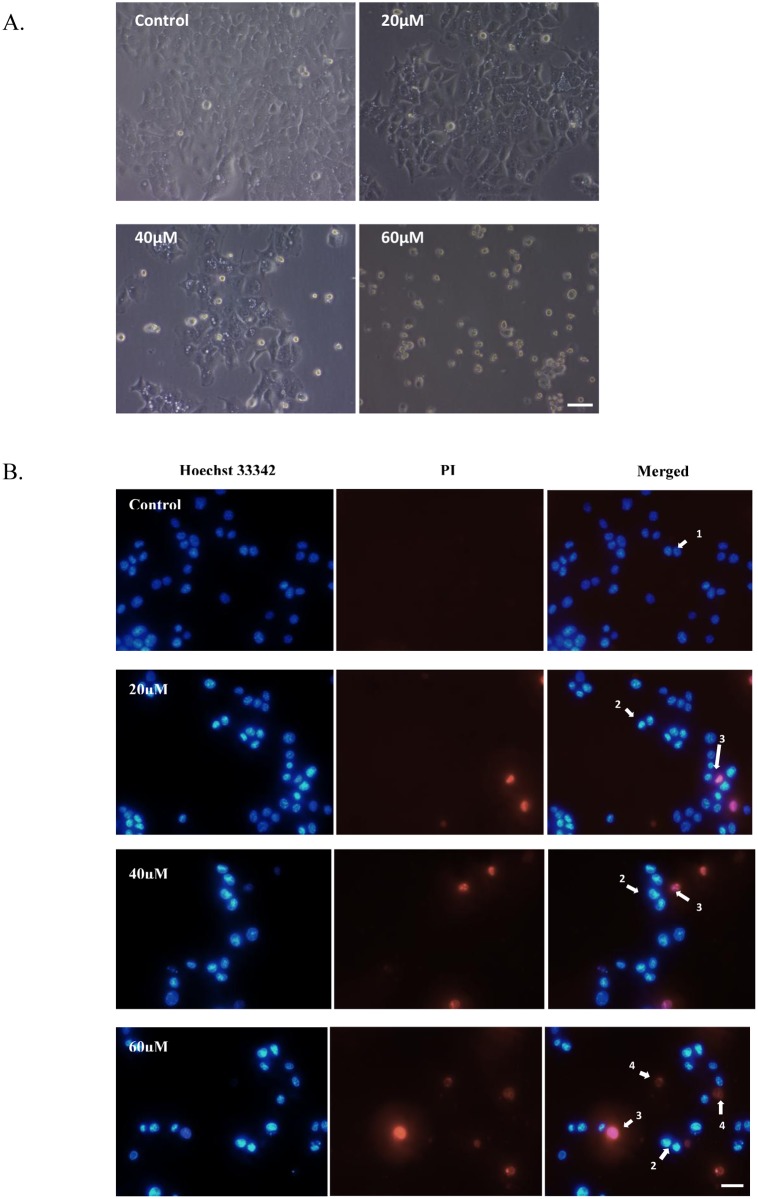
FKC induces apoptosis in HCT 116 cells. (A) HCT 116 cells were treated with FKC at the indicated concentrations for 48 hours, and subsequently observed under an inverted phase contrast microscope at magnification of 40× (Scale bar = 1μm). Control cells showing normal morphology (well spread and normal nuclei structure) while treated cells showing the typical morphological features of apoptosis include cell shrinkage, condensed or fragmented nuclei, membrane blebbing, vacuole formation and detachment of apoptotic bodies. (B) Representative fluorescence microscopy images of HCT 116 cells stained with Hoechst 34222 and PI after treated without or with the indicated concentrations of FKC for 48 hours and visualized using fluorescence microscope at magnification of 40× (Scale bar = 50μm). Arrows labelled with the following number and letter indicates: (1) viable cells with normal nuclei; (2) early apoptotic cells with highly condensed chromatin or fragmented chromatin (b); (3) late apoptotic cells with highly condensed chromatin or fragmented chromatin; (4) dead cells/necrosis. Untreated cells in 0.5% DMSO served as the control in (A) and (B).

To determine whether the cytotoxic effect of FKC was associated with the induction of apoptosis, morphological changes of cells were evaluated using phase-contrast microscopy. As shown in Fig 3A, control cells observed under phase-contrast microscopy were in tightly packed groups and retained the typical epithelial morphology. After treatment of HCT 116 cells with FKC at various concentrations for 48 hours, it was observed that cell numbers were reduced and the remaining cells displayed fewer cell to cell interactions, vacuolation in the cytoplasm, membrane blebbing, nuclear disintegration and formation of apoptotic bodies.

In order to distinguish between live, early or late apoptotic and necrotic cells, cells treated with FKC were evaluated by double staining with Hoechst 33342 and propidium iodide (PI) to examine the changes in nuclear morphology. Untreated cells showed a dull blue colour indicating healthy and viable cells. After treatment for 48 hours, a population of cells which showed bright blue and pink fluorescence with condensed or fragmented nuclei was observed indicating the presence of early and late apoptotic cells, respectively. It was observed that some cells were undergoing necrosis-like cell death after being treated with 60μM of FKC for 48 hours (red coloured). The results obtained thus far indicated that the cytotoxic effect of FKC on HCT 116 cells was associated with induction of apoptosis. Further experiments were necessary to validate the initial observation.

### FKC-induced apoptosis is associated with externalization of phosphatidylserine (PS) and DNA fragmentation in HCT 116 cells

**Fig 4:**
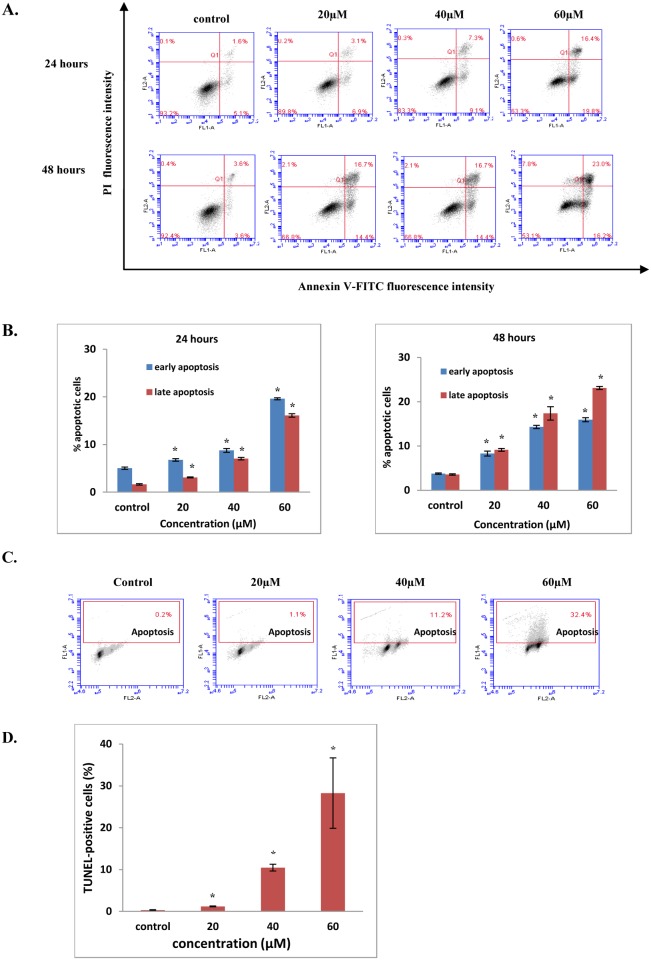
FKC induces phosphotidylserine (PS) externalization and DNA fragmentation in HCT 116 cells. (A) HCT 116 cells were treated with increasing concentrations of FKC for 48 hours and stained with AnnexinV-FITC and PI, followed by flow cytometric analysis. Untreated cells in 0.5% DMSO served as the control. Each dot plot is the representative result of three independent experiments. Cell populations are distinguished based on the four quadrants from a dot plot: viable (bottom left), early apoptotic (bottom right), late apoptotic (top right), and dead cells/debris (top left) cells. (B) Quantification of number of early and late apoptotic cells (from total 10,000 cells) of HCT 116 cells measured by flow cytometry for 24 and 48 hours are presented as percentages in bar charts. (C) Cells were treated with absence or presence of FKC at the indicated concentrations for 48 hours, and assessed using TUNEL assay kit. The percentages of cells with fragmented DNA were analyzed using flow cytometry as indicated in the upper quadrant from a dot plot. Each dot plot is the representative result of three independent experiments. (D) Percentages of HCT 116 cells which showed positive DNA fragmentation measured by flow cytometry are presented in bar diagram. Values given are expressed as mean±SD of triplicates obtained from three independent experiments. The asterisk (*) shows statistically significant differences in comparison to the control, p<0.05. Untreated cells in 0.5% DMSO served as the control.

Exposure of PS on the surface of apoptotic cells is a common marker for apoptosis and serve as a recognition signal for engulfment by phagocytes such as macrophages and dendritic cells and by their neighboring cells [30]. To further characterize the apoptotic features of HCT 116 cells after treatment with FKC, Annexin V-PI double staining was performed. It was found that there was a significant increase (p<0.05) in early and late apoptotic cells after treatment with increasing concentrations of FKC and incubation times in HCT 116 cells when compared to control (Fig 4A and 4B). After 24 hours incubation of HCT 116 cells with FKC, there was a higher increase in the percentage of early apoptotic cells compared to late apoptotic cells as the concentration of FKC was increased (Fig 4A and 4B). Extending the incubation period to 48 hours resulted in a greater increase in the percentage of late apoptotic cells compared to early apoptotic cells for all concentrations of FKC.

Internucleosomal DNA fragmentation is one of the last stages of apoptosis resulting from the activation of endogenous DNase, which cut the internucleosomal regions into double-stranded fragments of 180 to 200 base pairs [31]. As shown in Fig 4C, there was a concentration-dependent increase in the amount of apoptotic cells with fragmented DNA for both colon cancer cell lines following treatment with FKC for 48 hours. Compared to the control, there was a significant increase in the amount of TUNEL-positive cells with increasing concentrations of FKC as shown in Fig 4D. These results were consistent with the results obtained from Hoechst 33342/PI staining. This suggested that FKC caused DNA fragmentation in HCT 116 cells which may be associated with the induction of apoptosis.

### FKC induces intrinsic and extrinsic apoptosis by activating caspase-3, -8, -9 and death receptors

**Fig 5:**
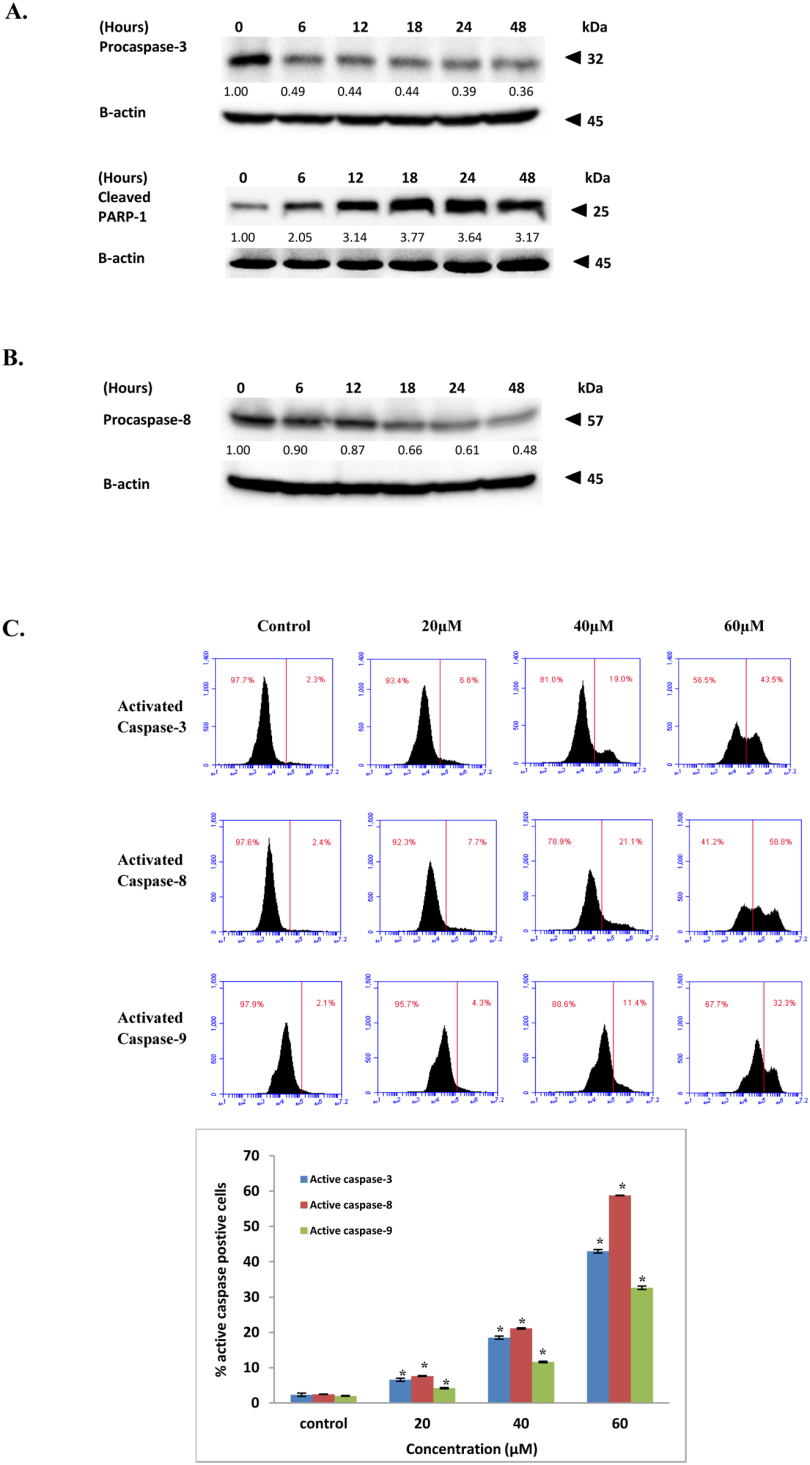
FKC induces activation of caspases and cleavage of PARP in HCT 116 cells. (A) and (B) Cells were treated with FKC (60μM) at the indicated time points. The levels of pro-caspase-3, -8 and cleaved PARP were analyzed by western blot and β-actin served as loading control. The results from representative experiments were expressed relative to the protein level at 0 hr after normalization to β-actin signals. (C) Activated caspase-3, -8 and -9 in HCT 116 cells were determined using flow cytometry. Cells were treated in the absence or presence of FKC at the indicated concentrations for 48 hours, and assessed using caspILLUME green Active caspase-3, -8 and -9 staining kit. The percentage of cells that showed positive for active caspase-3, -8 and -9 were quantified by flow cytometry and are presented in the bar chart. Values given are expressed as mean ± SD of triplicates obtained from three independent experiments. The asterisk (*) indicated p<0.05 when compared to the control. Untreated cells in 0.5% DMSO served as the control.

To determine whether the apparent induction of apoptosis was associated with the activation of caspase-3, -8 and -9, the presence of the different caspases were examined by western blotting and flow cytometry. Western blot analysis showed that the level of the procaspase-3 precursor was decreased in a time-dependent manner after FKC treatment, thereby suggesting cleavage and activation of the enzyme (Fig 5A). Similar results were observed for the procaspase-8 precursor (Fig 5B). Through flow cytometric analysis (Fig 5C), the percentages of activated caspase-3, -8 and -9 were found to have increased significantly in HCT 116 cells after treatment with increasing concentrations of FKC (20, 40 and 60 μM) in comparison to the control. Active caspase-8 was found to be generally more prominent than active caspase-9, thereby suggesting a preferential activation of the extrinsic versus intrinsic pathways of apoptosis after FKC treatment. Activation of caspase-3 was further confirmed by western blot analysis of the p25 fragment of poly-ADP-ribose polymerase (PARP) which results from the caspase-3 cleavage of the intact PARP (116 kDa) during apoptosis. Western blot analysis (Fig 5A) showed that exposure of HCT 116 cells to FKC increased the level of the 25 kDa fragment of PARP as compared to the control. The increased level of cleaved PARP was shown to be correlated with increased activation of caspase-3 in HCT 116 cells in this study. Taken together, these results showed that FKC activated caspase-8, -9 and -3 in a dose- and time-dependent manner.

Caspase-8 is involved in the extrinsic apoptosis pathway that occurs upon activation of cell surface receptors. We also investigated the involvement of death receptor mediated apoptotic pathway induced by FKC by evaluating the levels of death receptors, DR5 and DR4 in HCT 116 cells. Western blot analysis revealed that levels of DR5, and to a lesser extent DR4, are increased in a time-dependent manner after FKC treatment (S1 Fig). Our results suggest that the death receptors play a role in the extrinsic apoptosis induced by FKC. Thus these data demonstrated that FKC can induce apoptosis in HCT 116 cells through both the mitochondrial and death receptor apoptotic pathways.

### FKC causes loss of mitochondrial membrane potential in HCT 116 and release of apoptotic factors to cytosol

**Fig 6:**
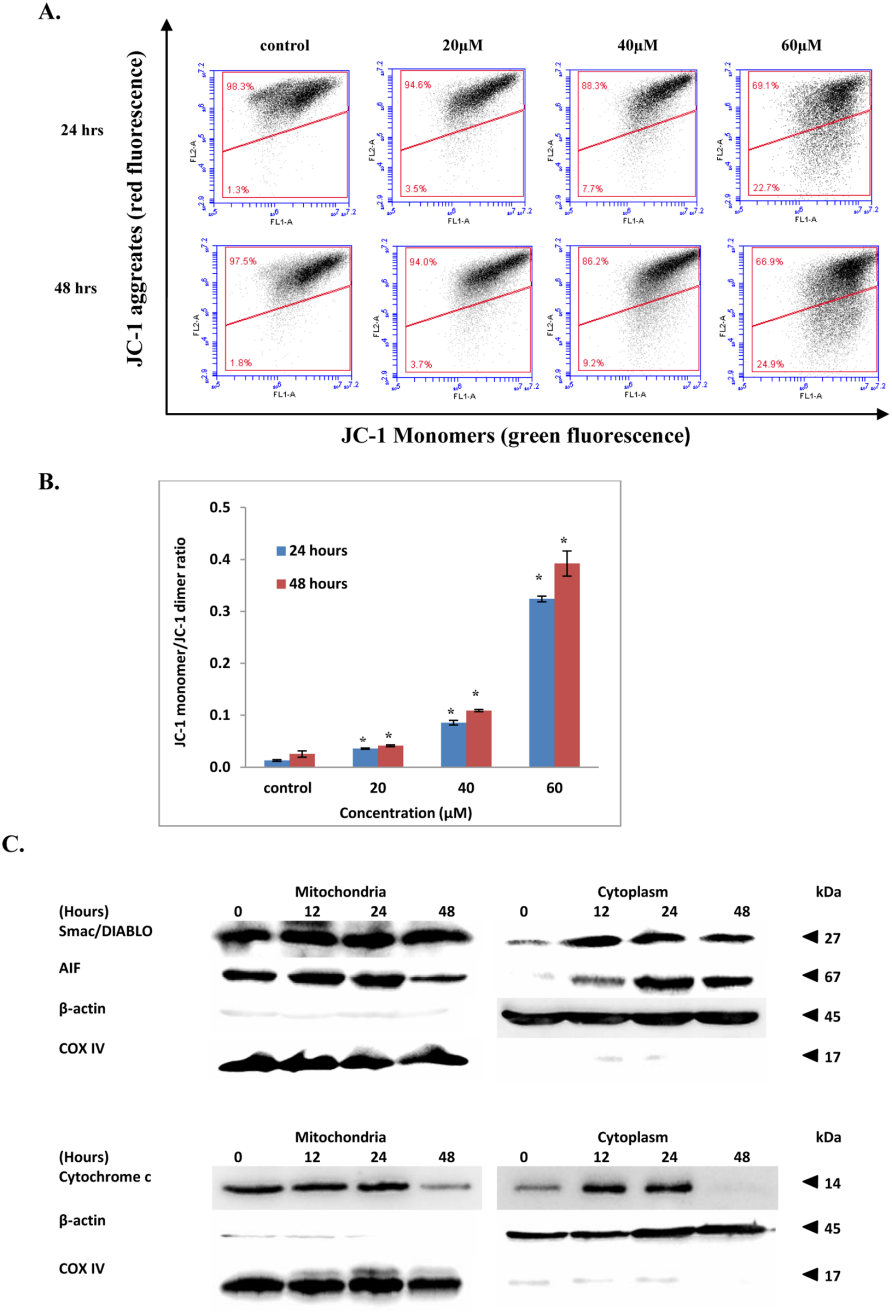
FKC causes dissipation of mitochondrial membrane potential in HCT 116 cells. (A) Cells were treated with the indicated concentrations of FKC for 48 hours. Cells were then incubated with JC-1 probe, and then analyzed using flow cytometry. Untreated cells in 0.5% DMSO served as the control. Upper quadrant indicates percentage of cells with polarized mitochondrial membranes which emit red fluorescence whereas bottom quadrant indicates percentages of cells with depolarized mitochondrial membranes which emit green fluorescence. Each dot plot is the representative result of three independent experiments. (B) The bar charts showing the ratio of mean intensity of JC-1 red fluorescence to JC-1 green fluorescence in HCT 116 cells treated with various concentrations for 24 and 48 hours. Values given are expressed as mean±SD of triplicates obtained from three independent experiments. The asterisk (*) indicated p<0.05 when compared to the control. (C) After treatment with FKC, whole cell lysate was fractionated into cytosolic and mitochondrial portions. Western blotting was used to examine the levels of cytochrome c, smac/DIABLO and AIF in both fractions of HCT 116 cells at indicated time points after FKC treatment (60μM). β-actin and COX IV were served as loading control for cytoplasm and mitochondria, respectively.

One important biochemical event in the intrinsic pathway which is indicative of early apoptosis is the sharp reduction in mitochondrial membrane potential due to an increase in the permeability of the mitochondrial membrane, leading to the release of apoptotic factors into the cytosol [32]. To ascertain the involvement of the mitochondrial pathway in the induction of apoptosis by FKC, the effect of FKC on the mitochondrial membrane potential (MMP) in HCT 116 cells was assessed using a fluorescent dye, JC-1 and analyzed by flow cytometry. A shift of fluorescent emission from red to green indicates a reduction in MMP. As shown in Fig 6A, an increase in green fluorescence of JC-1 monomers was observed in HCT 116 cells following a 48 hour treatment with various concentrations of FKC (20, 40 and 60μM) compared to control. As shown in the quantitative data of the ratio of dimer/monomer of JC-1 (Fig 6B), there was a significant increase (p<0.05) in ratio in HCT 116 cells treated with increasing concentrations of FKC for 24 and 48 hours compared to control. These findings suggested that exposure to FKC caused a significant change in mitochondrial membrane depolarisation in HCT 116 cells.

To determine whether the mitochondrial apoptotic pathway causes the translocation of pro-apoptotic proteins from mitochondria to cytosol, the concentrations of cytochrome c, apoptosis-inducing factor (AIF) and Smac/DIABLO present in the mitochondria and cytosol were examined using western blotting. The content of cytochrome c, AIF and Smac/Diablo was found to be gradually increased in the cytosol fractions after exposure to FKC, indicating that both proteins were released to the cytosol from the mitochondria (Fig 6C). These results suggested that FKC targeted the mitochondria causing a collapse in MMP.

### FKC-induced HCT 116 cells apoptosis is mediated by modulation of Bcl-2 family proteins and downregulation of anti-apoptotic proteins

Western blotting was performed to explore the potential role of the Bcl-2 family members in the regulation of the intrinsic and/or mitochondrial apoptotic pathway in HCT 116 cells treated with FKC. It was of particular interest to investigate whether the levels of anti-apoptotic and pro-apoptotic proteins was altered in HCT 116 cells after treatment with FKC. The level of the pro-apoptotic protein Bak was found to increase in HCT 116 cells in a time-dependent manner. However, the level of Bax was found to decrease after 18 hours of incubation with FKC. As shown in S1 Fig, the levels of the anti-apoptotic protein Bcl-2 were unaffected after the treatment. The level of Bcl-xL remained unaffected only in the first 18 hours and the level was markedly reduced after 18 hours of treatment (S1 Fig). The level of another pro-apoptotic protein, Bid was also examined, and it was found that there were no significant changes in its total protein level.

**Fig 7:**
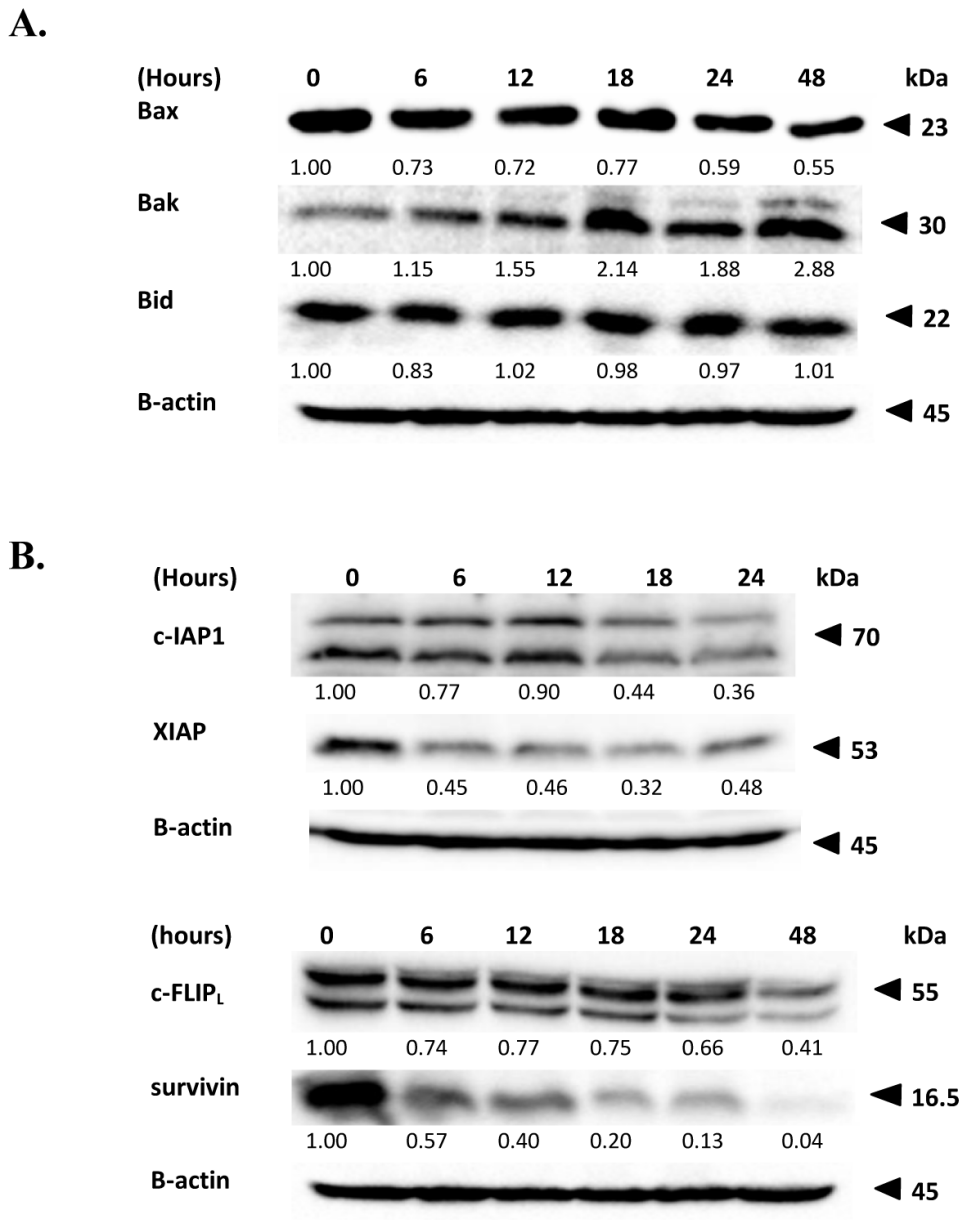
Effects of FKC on the levels of pro-apoptotic and anti-apopotic proteins in HCT 116 cells. HCT 116 cells were treated with FKC (60μM) for indicated time points, and followed by protein extraction and western blot analysis. (A) Effect of FKC on the levels of Bcl-2 family proteins in HCT 116 cells. (B) FKC inhibits the levels of c-IAP-1, XIAP, c-FLIP_L_ and survivin. The results from representative experiments were expressed relative to the protein level at 0 hr after normalization to β-actin signals.

We also investigated the levels of anti-apoptotic proteins, XIAP, cIAP-1, c-FLIP_L_ and survivin following FKC treatment. Western blot analysis (Fig 7B) showed that there was a dramatic decrease in the levels of XIAP and survivin in HCT 116 cells with increasing incubation time. The levels of c-IAP-1 and c-FLIPL were found to be downregulated after 12 hours of FKC treatment. Taken together, these findings suggested that the levels of pro-apoptotic proteins increased concurrently with a decrease in the levels of anti-apoptotic proteins in HCT 116 exposed to FKC.

### FKC induces cell cycle arrest of HCT 116 cells

**Fig 8:**
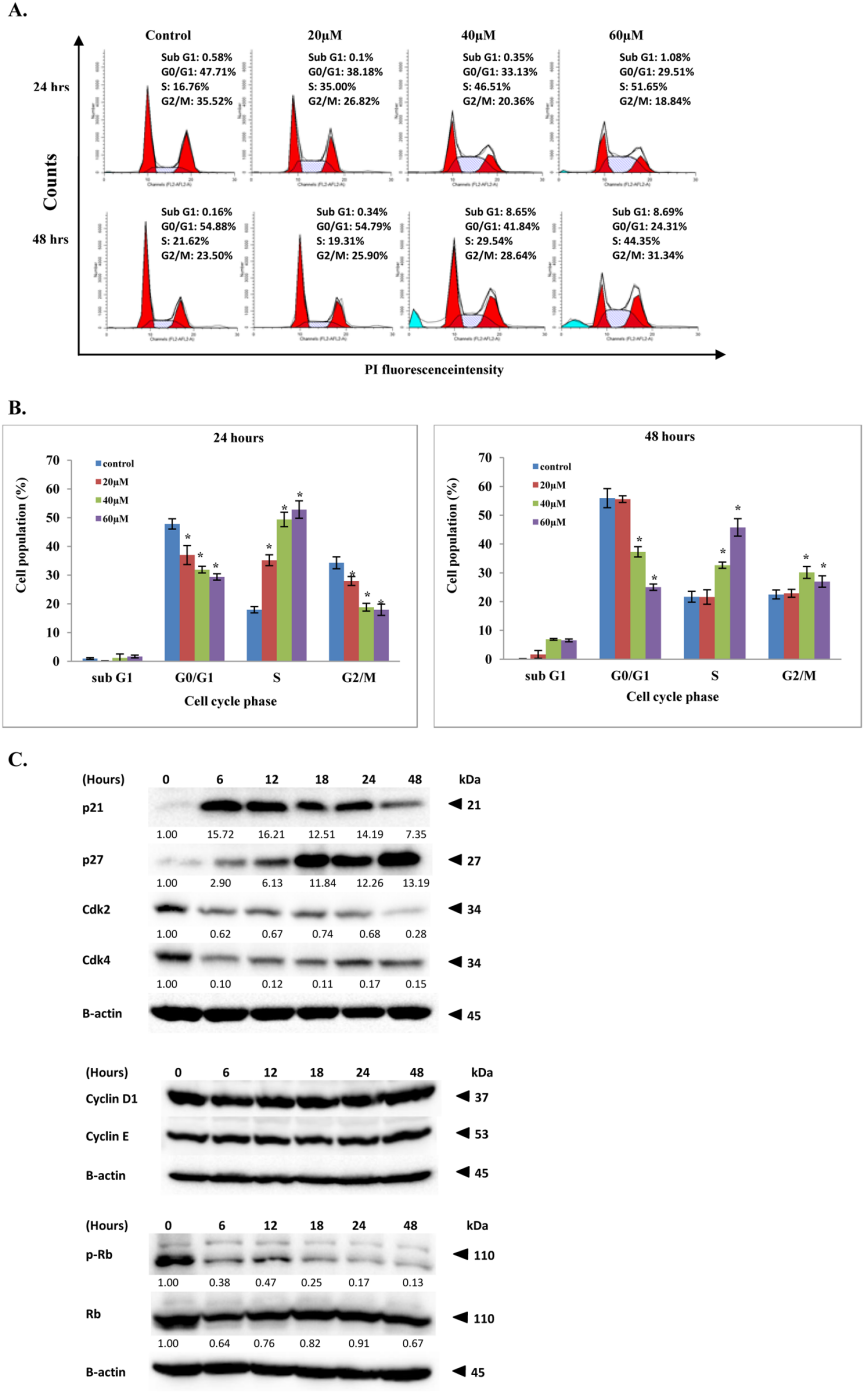
Effect of FKC on the cell cycle and the cell cycle regulatory proteins in HCT 116 cells. (A) Cells were treated with the indicated concentrations of FKC for 24 and 48 hours, and stained with PI. DNA content of HCT 116 was analyzed using flow cytometry and quantification of cell cycle distribution(sub-G_1_, G_0_/G_1_, S and G_2_/M phases)was performed using ModFit software. Untreated cells in 0.5% DMSO served as the control. Each histogram is the representative cell cycle profile of three independent experiments. (B) The quantitative data of mean of percentages of cells in each phase for HCT 116 cells for 24 and 48 hours are presented in bar chart. Values given are expressed as mean ± SD of triplicates obtained from three independent experiments. The asterisk (*) indicated *p*<0.05 when compared to the control. (C) Cells were incubated in the absence or presence of FKC (60μM) for the indicated times, and followed by protein extraction. Changes in levels of the cyclins (cyclin D1 and E), cyclin dependent kinase (Cdk2 and 4), Cdk inhibitors (p21^Cip1^ and p27^Kip1^), and RB phosphorylation were analyzed by Western blot. Untreated cells in 0.5% DMSO served as the control. β-actin was used as the loading control. The results from representative experiments were expressed relative to the proteins level at 0 hr after normalization to β-actin signals.

Cell proliferation is correlated with the regulation of cell cycle progression. An additional investigation was conducted to examine whether FKC trigger the molecular mechanisms underlying cell cycle arrest in HCT 116 cells. The cell cycles of HCT 116 cells were evaluated using flow cytometry with propidium iodide labelling to determine which phases of the cell cycle were arrested in cells treated with FKC. As shown in Fig 8A and 8B, there was an accumulation of cells in sub-G_1_ phase in HCT 116 cells treated with FKC after 48 hours incubation at 40 and 60 μM (8.65% and 8.69%, respectively) compared to the control. The percentage of cells in the S phase also increased significantly after FKC treatment for 24 and 48 hours accompanied by a decrease in the percentage of cells in the G_1_ phase. For instance, treatment of FKC at 40 and 60 μM for 24 hours significantly increased the percentages of cells in S phase (46.51% and 51.65%, respectively) compared to 16.76% in the control. We observed that treatment with FKC at 48 hours resulted in a slight increase in G_2_/M phase; however the induced S phase arrest was more apparent compared to G_2_/M arrest. These results indicated that there was a concentration- and time-dependent increase in the percentage of cells entering sub-G_1_ and S phases of the cell cycle in HCT 116 cells.

### Effect of FKC on the levels of cyclin/CDK, p21^Cip1^ and p27^Kip1^ in HCT 116 cells

To examine the mechanism responsible for cell cycle arrest induced by FKC, the effect of FKC on cell cycle regulatory proteins (cyclins), cyclin dependent kinases (Cdks) and cdk interacting protein/kinase inhibitory proteins (CIPs/KIPs) (which are involved in the regulation of the cell cycle progression) were evaluated by western blotting. As shown in Fig 8C, FKC treatment induced a dramatic decrease in the protein levels of Cdk2 and Cdk4 in HCT 116 cells. However, no changes in cyclin D1 and cyclin E were observed. Consistent with Cdks inhibition, the levels of cyclin-dependent kinase inhibitor, p21 and p27 were markedly upregulated following FKC treatment in HCT 116 cells. We also examined the effect of FKC on the phosphorylation status of Rb by western blotting. FKC markedly inhibited the phosphorylation of Rb (pRB) as early as 6 hours, with minimal changes in the level of total RB protein (Fig 8C). Based on these results, it can be summarized that FKC inhibited HCT 116 cell proliferation through upregulation of p21 and p27, downregulation of Cdk2 and Cdk4, and inhibition of RB phosphorylation, resulting S phase cell cycle arrest which subsequently led to cell death.

### FKC induced apoptosis through endoplasmic reticulum stress

**Fig 9:**
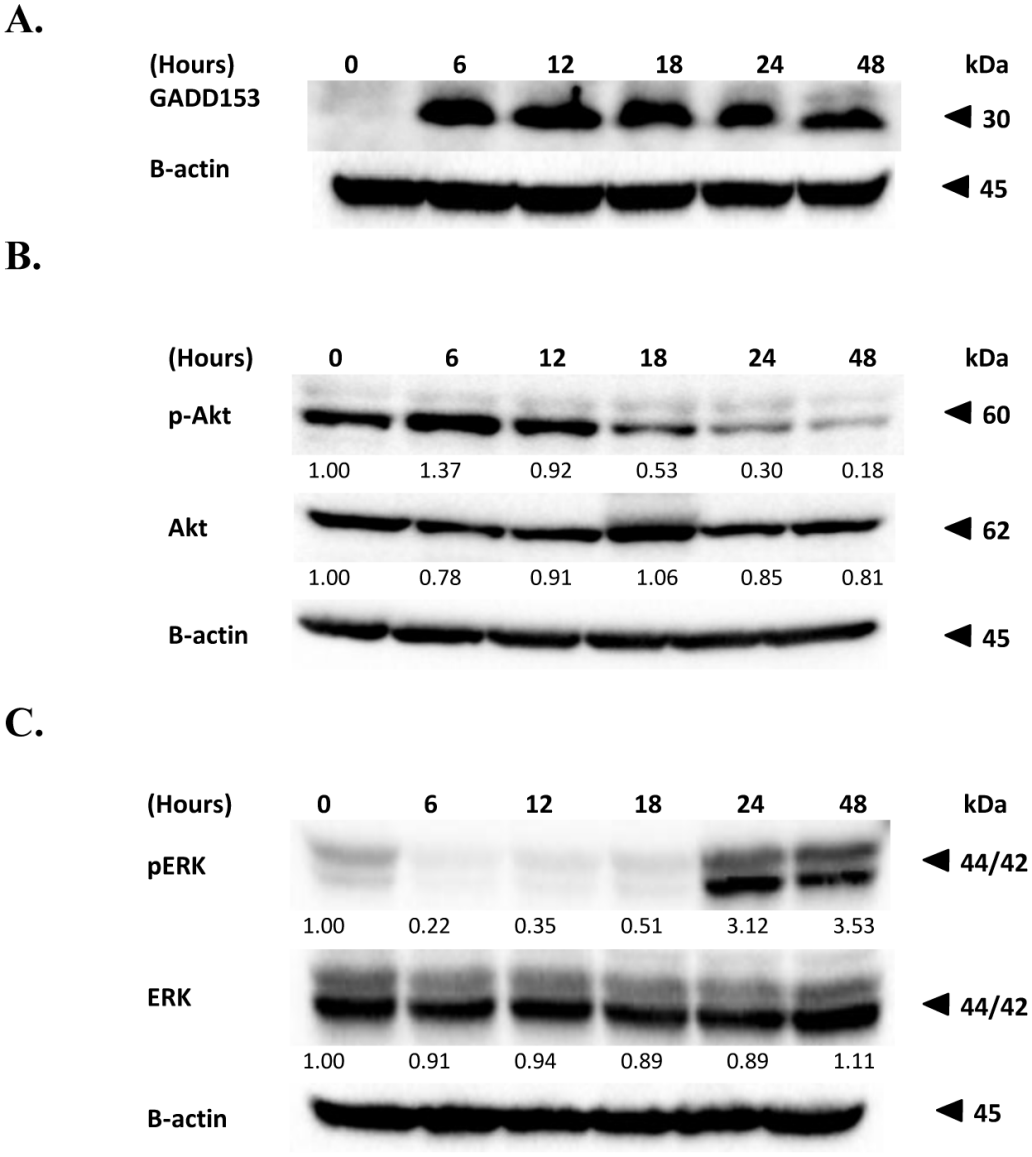
Effect of FKC on the protein levels involved in endoplasmic reticulum stress, MAPKs and Akt signaling pathways. (A) Cells were incubated with FKC (60μM) for the indicated time points and followed by protein extraction. The change in level of GADD153/CHOP were evaluated by western blot. (B) and (C) Cells were incubated with FKC (60μM) for the indicated time points and followed by protein extraction. Cell lysates were subjected to western blot analysis using antibodies indicated to detect the levels of phosphorylated or total proteins of ERK and Akt. Untreated cells in 0.5% DMSO served as the control. β-actin in the western blot was used as the internal control. The results from representative experiments were expressed relative to the proteins level at 0 hr after normalization to β-actin signals.

To further elucidate the possible apoptotic pathway triggered by FKC, we investigated the level of GADD153/CHOP using western blot analysis in HCT 116 cells treated with 60μM of FKC. The protein level of ER stress-associated molecules, GADD153 was investigated by western blotting. GADD153, also known as CHOP, encodes a member of the CCAAT/enhancer-binding protein family and acts as an inhibitor or activator of transcription, leading to apoptosis [33]. As shown in Fig 9A, it was observed that GADD153 was largely upregulated in HCT 116 cells after treatment with 60μM of FKC throughout the 48 hours incubation, while no expression of the protein was detected in non-treated cells. This implied that ER stress induced by FKC occurred in both intrinsic and extrinsic pathways in HCT 116 cells.

### Effect of FKC on MAPKs phosphorylation and Akt signaling pathways

MAPKs and Akt signaling pathways are known to be involved in cellular proliferation, survival and differentiation [34]. Therefore, western blot analysis was performed to investigate whether these signaling pathway were functionally involved in the apoptosis effect of FKC in HCT 116 cells after treatment with 60μM of FKC at different time intervals. The activation of Akt was detected using a phospho-specific Akt (Ser473) antibody. As shown in Fig 9B, it was found that there was a slight increase in Akt phosphorylation after 6 hours of treatment, after which the level of phosphorylation was gradually decreased while no apparent change was observed in total Akt under the same treatment condition.

We next investigated the effect of FKC on the activation of MAPKs cascade including extracellular regulated protein kinase (ERK), c-Jun N-terminal kinase (JNK) and p38 in HCT 116 cells. Western blot analysis (Fig 9C) revealed that FKC decreased the phosphorylation (Thr202/Tyr204) of ERK at the early time points (6, 12 and 18 hours post-FKC), whereas the drug caused dramatic increase in phosphorylation at later time points (24 and 48 hours post-FKC). No significant change in p38 phosphorylation was observed, whereas small reductions in JNK phosphorylation were noted at 24 and 48 hours post-FKC (-S1 Fig). The levels of total ERK, JNK and p38 protein remain unchanged after treatment with FKC. Together, these results suggested that apoptosis-induced FKC is involved in the inactivation of Akt pathway, and modulation of MAPKs pathway.

## Discussion

In the present study, the potential growth inhibitory and apoptosis-inducing effects of FKC on human cancer cell lines was explored. To the best of our knowledge, this is a first report to demonstrate the anti-proliferative and apoptosis inducing effect of FKC against HCT 116 carcinoma cancer cells while showing less cytotoxicity towards normal colon cells. In order to evaluate whether slight variations in the structure of FKC play an important role in cytotoxic and apoptotic activity, the effect of GMM (substitution at C-2’ and C-4, as shown in Fig 1) was investigated. Fig 1 show differences in the substitution at position C-2’ and C-4. FKC has a methoxyl group at C-2’ and a hydroxyl group at C-4 whilst GMM has a hydroxyl and methoxyl substituents at C-2’ and C-4 respectively. This change resulted in the absence of cytotoxic effect in all model cell lines (IC_50_>300 μM) in GMM. Thus, this suggested that reversing the substituents as in FKC resulted in pronounced cytotoxic activity of FKC in the HCT 116 cells. Based on previous studies, replacement of hydroxyl group at C-6’ of GMM with a methoxyl substituent as in flavokawain A (FKA) (Fig 1) resulted in pronounced cytotoxicity and FKA was able to induce apoptosis in bladder cells [13]. The absence of a functional group at aromatic ring B in FKB did not affect its apoptotic activity as FKB has been previously reported to cause cytotoxicity and induced apoptosis in HCT 116 cells [35–36]. Taken together, these results suggested that the cytotoxic and apoptotic activities of chalcones are clearly dependent on its molecular structure. Further work to understand the structure-activity relationship of this class of compounds is required.

In the intrinsic apoptotic pathway, the signal leading to cell death typically originates from within the cell itself. The mitochondria play a major role in the initiation and execution of the intrinsic pathway of apoptosis besides as sites of electron transport and generation of cellular ATP [37–38]. Bcl-2 family members are key regulators involved in controlling permeability of mitochondrial outer membrane permeabilization and causes leakage of apoptogenic proteins such as cytochrome c and other mitochondrial apoptotic factors like Smac/DIABLO, AIF and endoglycosidase G into the cytosol. Released cytochrome c binds to Apaf-1and pro-caspase-9 to form the apoptosome, which in turn activates caspase-9. In general, Bcl-2 related proteins are categorized into two groups: anti-apoptotic proteins (Bcl-2 and Bcl-xL), pro-apoptotic proteins (Bax, Bak and Bid) [39]. During apoptosis, Bax and Bak are known to be responsible for promoting mitochondrial outer membrane permeabilization by oligomerizing to form pores within the outer mitochondria membrane [39]. On other hand, Bcl-2 and Bcl-xL acts as the inhibitor of apoptosis by binding with pro-apoptotic proteins of the bcl-2 family and thus antagonizing them [17]. Therefore, they limit permeabilization of the mitochondrial outer membrane and maintain the mitochondria membrane potential by inhibiting pore formation [38, 40]. In addition, Bcl-xL can interact with Apaf-1 and inhibit the activation of capase-9 [41]. Therefore, the downregulation of Bcl-xL (S1 Fig) and an increase in the amount of Bak (Fig 7) in HCT 116 cells upon FKC treatment may have caused a disruption in the integrity of the outer mitochondria membrane by increasing its permeability, resulting in the release of cytochrome c, Smac/DIABLO and AIF from the outer membrane of mitochondria into the cytosol (Fig 6C). A previous study reported that Bak deficiency can lead to substantial inhibition of mitochondrial-mediated apoptotic cell death [42]. The western blot analysis in this study demonstrated the gradual increase in the concentration of AIF and Smac/DIABLO in the cytosol fraction upon FKC treatment. AIF is normally present in the mitochondria and will translocate into the nucleus following the release from mitochondria where it induces caspase-independent chromatin condensation and DNA fragmentation [42–43].

In the extrinsic pathway, apoptosis is initiated through the binding of cognate ligands to the respective death receptors. This will lead to the recruitment of adaptor molecules such as Fas-associated death domain protein (FADD) and TNF receptor-associated death doman protein (TRADD) through their complementary death domains which will then bind to procaspase-8. A death-inducing signaling complex (DISC) is formed, resulting in dimerization and activation of caspase-8. The activated caspase-8 directly cleave and activate caspase-3 [44]. However, cellular FLICE-like inhibitory protein (c-FLIP) competes with pro-caspase-8 to bind to the FADD in the DISC formation process [45]. Thus they block the activation of caspase-8 which in turn inhibits the activation of apoptosis triggered by death receptors. Based on our findings, we propose that FKC activate the extrinsic pathway by increasing the levels of DR5, and to a lesser extent DR4, and down-regulation of c-FLIP_L_. These result was consistent with the higher amount of active caspase-8 detected in comparison to active caspase-9 in HCT 116 cells upon FKC treatment in dose-dependent manner. A link with the mitochondrial pathway exists via caspase 8-mediated cleavage of Bid in which the truncated Bid migrates to the mitochondria and activates the pro-apoptotic members Bak and Bax. Western blot analysis was used to examine the level of Bid. However, no changes were observed in the level of Bid after FKC treatment, suggesting that Bid was not involved in the FKC-induced apoptosis.

The human inhibitor of apoptosis protein (IAP) family members consist of eight proteins which contain either one or three Baculovirus IAP Repeat (BIR) domain [46]. Among the inhibitor of apoptosis proteins, XIAP, cIAP-1, cIAP-2, ML-IAP and survivin are endogenous caspase inhibitors that inhibit apoptosis and lead to cell survival while others are involved in cell cycle and inflammation [46]. XIAP are the most potent caspase inhibitor as it directly binds and inhibits the caspases activation. XIAP binds caspase-9 through its BIR3 domain and preventing its dimerization. It also binds to activated caspase-3/7 through its BIR2 domain and the linker region between the BIR1 and BIR2 domains. However, XIAP can be inhibited by Smac/DIABLO which is released into the cytosol from the mitochondria upon loss of outer mitochondrial membrane potential. The released Smac/DIABLO binds to XIAP via its IAP-binding motif, and promote their auto-ubiquitination and consequent degradation [47]. In contrast, cIAP-1 indirectly inhibits caspase-3/8 activation through its E3 ligase activity as well as interaction with the TNR receptor-associated factor 1 and 2 (TRAF1 and TRAF2) [48]. Survivin, the smallest member of the IAP family of proteins, has been found to be highly expressed in tumors and associated with a metastatic phenotype, shorter survival times, and a resistance to chemotherapy in patients. Survivin indirectly inhibits caspase-9 activation by binding to Smac/DIABLO, thus preventing it from binding to XIAP [49]. In addition, inhibition of XIAP can be caused by binding to Smac/DIABLO which was found to be released into the cytoplasm upon FKC treatment. Based on the western blot results, the levels of cIAP-1, XIAP and survivin were dramatically decreased after FKC treatment and this subsequently resulted in the activation of caspase-8, -9 and -3.

Deregulation of the cell cycle is one of the hallmarks of tumorigenesis and contributes to the uncontrolled proliferation in human cancer. Molecular mechanisms of cell cycle regulation in cancer is disrupted by mutations in key checkpoint genes [50]. The phases of the cell cycle can be divided into four in which the periods of DNA synthesis (S phase) and mitosis (M phase) is separated by gaps called G_1_ and G_2_ [51]. Transition from one phase to another of the cell cycle is regulated by a series of checkpoint involving a family of cyclin-dependent kinases (CDKs) through binding with their respective regulatory subunits (cyclins), which then trigger different downstream processes of the cycle by phosphorylating appropriate target proteins [52]. The activities of CDK-cyclin complexes are negatively regulated by the endogenous CDK inhibitors, p21^Cip^ and p27^Kip^ or degradation of cyclin [52]. p21 and p27 inhibits and binds to Cdk4/cyclin D and Cdk2/cyclin E complexes respectively, resulting in de-phosphorylation of retinoblastoma (RB) proteins and inhibiting the release of E2F transcription factor into the nucleus to activate the transcription of cell cycle related genes [53–54]. Another complexes, Cdk2/cyclin E plays a critical role in promoting progression through S phase, along with additional phosphorylation of retinoblastoma [55]. Blockade of DNA synthesis in S phase may prevent the replication of the damaged or mutated DNA which allows the cells to either repair DNA damage before entering mitosis or undergo apoptosis [56]. One possible explanation for the inhibitory effect of FKC on cell cycle arrest was that it was due to the reduction of Cdk2, Cdk4 and upregulation of p21^Cip^ and p27^Kip^. The S phase arrest was also correlated with the de-phosphorylation of Rb.

Targeting endoplasmic reticulum (ER) stress has received a great deal of attention as the molecular pathway that can lead to the cell death through apoptosis as well as increased the sensitivity of tumor towards chemotherapeutic agents [57]. The ER plays an essential role in the proper folding and posttranslational modification of secreted and membrane proteins, maintenance of calcium homeostasis and lipid biosynthesis [58]. Accumulation of unfolded proteins and disturbance of calcium homeostasis within ER causes ER stress. Prolonged or severe ER stress can result in apoptosis via intrinsic or extrinsic-mediated pathways to eliminate the damaged cells [58–59]. GADD153/CHOP is a key factor in ER stress-induced apoptosis in which the increased level of CHOP can induce the transcription of various genes that activate the apoptotic pathways, which involves inhibition of Bcl-2 and stimulation of DR5, activation of caspases, increased outer mitochondrial membrane permeabilization and amplification of death signals [60]. Western blot results showed the increase in the level of CHOP as early as 6 hours after FKC treatment which suggested the occurrence of endoplasmic reticulum stress in HCT 116 cells, leading to apoptotic cell death.

Many studies have reported the possible interlink between the Akt/PI3K and MAPKs pathways in the regulation of cell proliferation and apoptosis. Therefore, in this study, we investigated the involvement of ERK1/2, p38 and JNK, and Akt pathways in the mechanism underlying the apoptotic properties of FKC. Interestingly, we found that FKC inhibited activation of Akt and this resulted in a dramatic increase in ERK1/2 phosphorylation (a 3-fold increase after 18 hours over the control) while a decrease in JNK phosphorylation. Our data also showed that p38 MAPK was also activated to a slight degree by FKC. However, activation of Akt was found to occur in HCT 116 cells following FKC treatment at the beginning of 6 hours. This phenomenon might be due to the transient response of the cells to an apoptotic stimulus as a self-defense mechanism to protect cells against apoptosis. It was noticed that the inhibition of Akt and activation of ERK1/2 occurred at a relatively late event in the response of HCT 116 cells to the FKC treatment (Fig 9A and 9B). These results suggests that there is an opposite regulation between Akt and ERK signaling pathways while a positive correlation between Akt and JNK phosphorylation in FKC-induced apoptosis. However, the mechanisms that can modulate both the Akt and MAPKs pathways in response to treatment with FKC remain unclear. The activation of the ERK1/2 pathway is normally thought to be associated with cell proliferation and survival. However, many studies have shown that ERK1/2 can exert a dual effect on cell growth. The anti-apoptotic effect of ERK1/2 activation has been shown to stimulate proliferation by increasing expression of cyclin D and inactivating p27 [61–62]. Activation of ERK1/2 has also been shown to be required for the induction of apoptosis by DNA-damaging agents such as doxorubicin and cisplatin which is accompanied by inactivation of Akt [22, 63]. Activation of ERK1/2 has been shown to induce apoptosis in T-cells via increasing Fas ligand expression [64]. ERK pathway is also involved in activating mitochondrial-dependent pathway through regulation of Bcl family proteins as well as extrinsic pathway by increasing the expression of ligands and death receptors [65]. Collectively, our results suggested that there is an interplay between the Akt signaling pathway and MAPKs pathway in induction of apoptosis and cell cycle arrest by FKC in HCT 116 cells.

## Conclusion

Our results provided evidence that FKC induced apoptosis in HCT 116 colon carcinoma through the intrinsic and extrinsic pathways. In addition, exposure to FKC induced cell cycle arrest at the S phase in HCT 116 cells, leading to growth inhibition followed by apoptosis. This event was associated with increased endoplasmic reticulum stress, inhibition of Akt activation and elevation of ERK activation. These findings provide fundamental insights into the molecular mechanism of FKC-induced cell death. This compound shows good promise and merits to be further evaluated as a potential therapeutic agent for the treatment of human HCT colon carcinoma.

## Supporting Information

S1 Fig Effects of FKC on the changes in levels of cytochrome c, DR5, DR4, Bcl-xL, Bcl-2, p-JNK, JNK, p-p38 and p38 in HCT 116 cells. HCT 116 cells were treated with FKC (60μM) for indicated time points, and followed by protein extraction and western blot analysis. The results from representative experiments were expressed relative to the proteins level at 0hr after normalization to β-actin signals. Further validation will be needed to confirm the effects of FKC on these proteins.
(TIF)

## References

[1] Ferlay J, Shin HR, Bray F, Forman D, Mathers C, Parkin DM. Estimates of worldwide burden of cancer in 2008: GLOBOCAN 2008. Int J Cancer. 2010;127(12):2893–917. 10.1002/ijc.25516 21351269

[2] Jemal A, Center MM, DeSantis C, Ward EM. Global patterns of cancer incidence and mortality rates and trends. Cancer Epidemiol Biomarkers Prev. 2010;19(8):1893–907. 10.1158/1055-9965.EPI-10-0437 20647400

[3] Aizat AA, Shahpudin SN, Mustapha MA, Zakaria Z, Sidek AS, Abu Hassan MR, et al Association of Arg72Pro of P53 polymorphism with colorectal cancer susceptibility risk in Malaysian population. Asian Pac J Cancer Prev. 2011;12(11):2909–13. 22393962

[4] Ng SC, Wong SH. Colorectal cancer screening in Asia. Br Med Bull. 2013;105:29–42. 10.1093/bmb/lds040 23299409

[5] Wan Puteh SE, Saad NM, Aljunid SM, Abdul Manaf MR, Sulong S, Sagap I, et al Quality of life in Malaysian colorectal cancer patients. Asia Pac Psychiatry. 2013;5 Suppl 1:110–7. 10.1111/appy.12055 23857846

[6] Vogel S, Ohmayer S, Brunner G, Heilmann J. Natural and non-natural prenylated chalcones: synthesis, cytotoxicity and anti-oxidative activity. Bioorg Med Chem. 2008;16(8):4286–93. 10.1016/j.bmc.2008.02.079 18343123

[7] Abu N, Ho WY, Yeap SK, Akhtar MN, Abdullah MP, Omar AR, et al The flavokawains: uprising medicinal chalcones. Cancer Cell Int. 2013;13(1):102 10.1186/1475-2867-13-102 24148263 PMC4015927

[8] Ho YF, Karsani SA, Yong WK, Abd Malek SN. Induction of apoptosis and cell cycle blockade by helichrysetin in a549 human lung adenocarcinoma cells. Evid Based Complement Alternat Med. 2013;2013:857257 10.1155/2013/857257 23533528 PMC3603683

[9] Dharmaratne HR, Nanayakkara NP, Khan IA. Kavalactones from Piper methysticum, and their 13C NMR spectroscopic analyses. Phytochemistry. 2002;59(4):429–33. 11830162 10.1016/s0031-9422(01)00443-5

[10] Cote CS, Kor C, Cohen J, Auclair K. Composition and biological activity of traditional and commercial kava extracts. Biochem Biophys Res Commun. 2004;322(1):147–52. 15313185 10.1016/j.bbrc.2004.07.093

[11] Whitton PA, Lau A, Salisbury A, Whitehouse J, Evans CS. Kava lactones and the kava-kava controversy. Phytochemistry. 2003;64(3):673–9. 13679089 10.1016/s0031-9422(03)00381-9

[12] Weiss J, Sauer A, Frank A, Unger M. Extracts and kavalactones of Piper methysticum G. Forst (kava-kava) inhibit P-glycoprotein in vitro. Drug Metab Dispos. 2005;33(11):1580–3. 16051732 10.1124/dmd.105.005892

[13] Zi X, Simoneau AR. Flavokawain A, a novel chalcone from kava extract, induces apoptosis in bladder cancer cells by involvement of Bax protein-dependent and mitochondria-dependent apoptotic pathway and suppresses tumor growth in mice. Cancer Res. 2005;65(8):3479–86. 15833884 10.1158/0008-5472.CAN-04-3803

[14] Li N, Liu JH, Zhang J, Yu BY. Comparative evaluation of cytotoxicity and antioxidative activity of 20 flavonoids. J Agric Food Chem. 2008;56(10):3876–83. 10.1021/jf073520n 18433100

[15] Ghobrial IM, Witzig TE, Adjei AA. Targeting apoptosis pathways in cancer therapy. CA Cancer J Clin. 2005;55(3):178–94. 15890640 10.3322/canjclin.55.3.178

[16] Wong RS. Apoptosis in cancer: from pathogenesis to treatment. J Exp Clin Cancer Res. 2011;30:87 10.1186/1756-9966-30-87 21943236 PMC3197541

[17] Tamm I, Schriever F, Dorken B. Apoptosis: implications of basic research for clinical oncology. Lancet Oncol. 2001;2(1):33–42. 11905616 10.1016/S1470-2045(00)00193-5

[18] Majors BS, Betenbaugh MJ, Chiang GG. Links between metabolism and apoptosis in mammalian cells: applications for anti-apoptosis engineering. Metab Eng. 2007;9(4):317–26. 17611135 10.1016/j.ymben.2007.05.003

[19] Tzifi F, Economopoulou C, Gourgiotis D, Ardavanis A, Papageorgiou S, Scorilas A. The Role of BCL2 Family of Apoptosis Regulator Proteins in Acute and Chronic Leukemias. Adv Hematol. 2012;2012:524308 10.1155/2012/524308 21941553 PMC3173728

[20] Elmore S. Apoptosis: a review of programmed cell death. Toxicol Pathol. 2007;35(4):495–516. 17562483 10.1080/01926230701320337 PMC2117903

[21] Lin FM, Tsai CH, Yang YC, Tu WC, Chen LR, Liang YS, et al A novel diterpene suppresses CWR22Rv1 tumor growth in vivo through antiproliferation and proapoptosis. Cancer Res. 2008;68(16):6634–42. 10.1158/0008-5472.CAN-08-0635 18701487

[22] Lee ER, Kim JY, Kang YJ, Ahn JY, Kim JH, Kim BW, et al Interplay between PI3K/Akt and MAPK signaling pathways in DNA-damaging drug-induced apoptosis. Biochim Biophys Acta. 2006;1763(9):958–68. 16905201 10.1016/j.bbamcr.2006.06.006

[23] Itoh N, Semba S, Ito M, Takeda H, Kawata S, Yamakawa M. Phosphorylation of Akt/PKB is required for suppression of cancer cell apoptosis and tumor progression in human colorectal carcinoma. Cancer. 2002;94(12):3127–34. 12115344 10.1002/cncr.10591

[24] Zhang W, Liu HT. MAPK signal pathways in the regulation of cell proliferation in mammalian cells. Cell Res. 2002;12(1):9–18. 11942415 10.1038/sj.cr.7290105

[25] Houghton P, Fang R, Techatanawat I, Steventon G, Hylands PJ, Lee CC. The sulphorhodamine (SRB) assay and other approaches to testing plant extracts and derived compounds for activities related to reputed anticancer activity. Methods. 2007;42(4):377–87. 17560325 10.1016/j.ymeth.2007.01.003

[26] Karmakar S, Banik NL, Patel SJ, Ray SK. Garlic compounds induced calpain and intrinsic caspase cascade for apoptosis in human malignant neuroblastoma SH-SY5Y cells. Apoptosis. 2007;12(4):671–84. 17219050 10.1007/s10495-006-0024-x

[27] Syed Abdul Rahman SN, Abdul Wahab N, Abd Malek SN. In Vitro Morphological Assessment of Apoptosis Induced by Antiproliferative Constituents from the Rhizomes of Curcuma zedoaria. Evidence-Based Complementary and Alternative Medicine. 2013;2013:14. 10.1155/2013/257108 PMC3671673 23762112

[28] Klamt F, Shacter E. Taurine chloramine, an oxidant derived from neutrophils, induces apoptosis in human B lymphoma cells through mitochondrial damage. J Biol Chem. 2005;280(22):21346–52. 15799967 10.1074/jbc.M501170200

[29] Chen SP, Yang HL, Her GM, Lin HY, Jeng MF, Wu JL, et al Betanodavirus induces phosphatidylserine exposure and loss of mitochondrial membrane potential in secondary necrotic cells, both of which are blocked by bongkrekic acid. Virology. 2006;347(2):379–91. 16430940 10.1016/j.virol.2005.11.052

[30] Elliott MR, Ravichandran KS. Clearance of apoptotic cells: implications in health and disease. J Cell Biol. 2010;189(7):1059–70. 10.1083/jcb.201004096 20584912 PMC2894449

[31] Darzynkiewicz Z, Galkowski D, Zhao H. Analysis of apoptosis by cytometry using TUNEL assay. Methods. 2008;44(3):250–4. 10.1016/j.ymeth.2007.11.008 18314056 PMC2295206

[32] Tait SW, Green DR. Mitochondria and cell death: outer membrane permeabilization and beyond. Nat Rev Mol Cell Biol. 2010;11(9):621–32. 10.1038/nrm2952 20683470

[33] Yamaguchi H, Wang HG. CHOP is involved in endoplasmic reticulum stress-induced apoptosis by enhancing DR5 expression in human carcinoma cells. J Biol Chem. 2004;279(44):45495–502. 15322075 10.1074/jbc.M406933200

[34] Johnson TL, Lai MB, Lai JC, Bhushan A. Inhibition of Cell Proliferation and MAP Kinase and Akt Pathways in Oral Squamous cell Carcinoma by Genistein and Biochanin A. Evid Based Complement Alternat Med. 2010;7(3):351–8. 10.1093/ecam/nen011 18955325 PMC2887331

[35] Kuo YF, Su YZ, Tseng YH, Wang SY, Wang HM, Chueh PJ. Flavokawain B, a novel chalcone from Alpinia pricei Hayata with potent apoptotic activity: Involvement of ROS and GADD153 upstream of mitochondria-dependent apoptosis in HCT116 cells. Free Radic Biol Med. 2010;49(2):214–26. 10.1016/j.freeradbiomed.2010.04.005 20398749

[36] Malek SN, Phang CW, Ibrahim H, Norhanom AW, Sim KS. Phytochemical and cytotoxic investigations of Alpinia mutica rhizomes. Molecules. 2011;16(1):583–9. 10.3390/molecules16010583 21240148 PMC6259141

[37] Zeestraten EC, Benard A, Reimers MS, Schouten PC, Liefers GJ, van de Velde CJ, et al The Prognostic Value of the Apoptosis Pathway in Colorectal Cancer: A Review of the Literature on Biomarkers Identified by Immunohistochemistry. Biomark Cancer. 2013;5:13–29. 10.4137/BIC.S11475 24179395 PMC3791955

[38] Harris MH, Thompson CB. The role of the Bcl-2 family in the regulation of outer mitochondrial membrane permeability. Cell Death Differ. 2000;7(12):1182–91. 11175255 10.1038/sj.cdd.4400781

[39] Lindsay J, Esposti MD, Gilmore AP. Bcl-2 proteins and mitochondria—specificity in membrane targeting for death. Biochim Biophys Acta. 2011;1813(4):532–9. 21056595 10.1016/j.bbamcr.2010.10.017

[40] Jain MV, Paczulla AM, Klonisch T, Dimgba FN, Rao SB, Roberg K, et al Interconnections between apoptotic, autophagic and necrotic pathways: implications for cancer therapy development. J Cell Mol Med. 2013;17(1):12–29. 10.1111/jcmm.12001 23301705 PMC3823134

[41] Hu Y, Benedict MA, Wu D, Inohara N, Nunez G. Bcl-XL interacts with Apaf-1 and inhibits Apaf-1-dependent caspase-9 activation. Proc Natl Acad Sci U S A. 1998;95(8):4386–91. 9539746 10.1073/pnas.95.8.4386 PMC22498

[42] Indran IR, Tufo G, Pervaiz S, Brenner C. Recent advances in apoptosis, mitochondria and drug resistance in cancer cells. Biochim Biophys Acta. 2011;1807(6):735–45. 10.1016/j.bbabio.2011.03.010 21453675

[43] Hu W, Kavanagh JJ. Anticancer therapy targeting the apoptotic pathway. Lancet Oncol. 2003;4(12):721–9. 14662428 10.1016/s1470-2045(03)01277-4

[44] Parrish AB, Freel CD, Kornbluth S. Cellular mechanisms controlling caspase activation and function. Cold Spring Harb Perspect Biol. 2013;5(6). 10.1101/cshperspect.a008672 PMC3660825 23732469

[45] Krueger A, Baumann S, Krammer PH, Kirchhoff S. FLICE-inhibitory proteins: regulators of death receptor-mediated apoptosis. Mol Cell Biol. 2001;21(24):8247–54. 11713262 10.1128/MCB.21.24.8247-8254.2001 PMC99990

[46] Berthelet J, Dubrez L. Regulation of Apoptosis by Inhibitors of Apoptosis (IAPs). Cells. 2013;2(1):163–87. 10.3390/cells2010163 24709650 PMC3972657

[47] Li P, Nijhawan D, Budihardjo I, Srinivasula SM, Ahmad M, Alnemri ES, et al Cytochrome c and dATP-dependent formation of Apaf-1/caspase-9 complex initiates an apoptotic protease cascade. Cell. 1997;91(4):479–89. 9390557 10.1016/s0092-8674(00)80434-1

[48] Guicciardi ME, Mott JL, Bronk SF, Kurita S, Fingas CD, Gores GJ. Cellular inhibitor of apoptosis 1 (cIAP-1) degradation by caspase 8 during TNF-related apoptosis-inducing ligand (TRAIL)-induced apoptosis. Exp Cell Res. 2011;317(1):107–16. 20951133 10.1016/j.yexcr.2010.10.005 PMC2991414

[49] Johnson ME, Howerth EW. Survivin: a bifunctional inhibitor of apoptosis protein. Vet Pathol. 2004;41(6):599–607. 15557069 10.1354/vp.41-6-599

[50] Novak B, Tyson JJ. Modelling the controls of the eukaryotic cell cycle. Biochem Soc Trans. 2003;31(Pt 6):1526–9. 14641104 10.1042/bst0311526

[51] Han CR, Jun do Y, Woo HJ, Jeong SY, Woo MH, Kim YH. Induction of microtubule-damage, mitotic arrest, Bcl-2 phosphorylation, Bak activation, and mitochondria-dependent caspase cascade is involved in human Jurkat T-cell apoptosis by aruncin B from Aruncus dioicus var. kamtschaticus. Bioorg Med Chem Lett. 2012;22(2):945–53. 10.1016/j.bmcl.2011.12.023 22197393

[52] Vermeulen K, Van Bockstaele DR, Berneman ZN. The cell cycle: a review of regulation, deregulation and therapeutic targets in cancer. Cell Prolif. 2003;36(3):131–49. 12814430 10.1046/j.1365-2184.2003.00266.x PMC6496723

[53] Giacinti C, Giordano A. RB and cell cycle progression. Oncogene. 2006;25(38):5220–7. 16936740 10.1038/sj.onc.1209615

[54] Munoz-Alonso MJ, Acosta JC, Richard C, Delgado MD, Sedivy J, Leon J. p21Cip1 and p27Kip1 induce distinct cell cycle effects and differentiation programs in myeloid leukemia cells. J Biol Chem. 2005;280(18):18120–9. 15746092 10.1074/jbc.M500758200

[55] Hinds PW, Mittnacht S, Dulic V, Arnold A, Reed SI, Weinberg RA. Regulation of retinoblastoma protein functions by ectopic expression of human cyclins. Cell. 1992;70(6):993–1006. 1388095 10.1016/0092-8674(92)90249-c

[56] Agarwal ML, Agarwal A, Taylor WR, Chernova O, Sharma Y, Stark GR. A p53-dependent S-phase checkpoint helps to protect cells from DNA damage in response to starvation for pyrimidine nucleotides. Proc Natl Acad Sci U S A. 1998;95(25):14775–80. 9843965 10.1073/pnas.95.25.14775 PMC24525

[57] Selimovic D, Ahmad M, El-Khattouti A, Hannig M, Haikel Y, Hassan M. Apoptosis-related protein-2 triggers melanoma cell death by a mechanism including both endoplasmic reticulum stress and mitochondrial dysregulation. Carcinogenesis. 2011;32(8):1268–78. 10.1093/carcin/bgr112 21693538

[58] Jin HR, Liao Y, Li X, Zhang Z, Zhao J, Wang CZ, et al Anticancer compound Oplopantriol A kills cancer cells through inducing ER stress and BH3 proteins Bim and Noxa. Cell Death Dis. 2014;5:e1190 10.1038/cddis.2014.169 24763047 PMC4001317

[59] McGuckin MA, Eri RD, Das I, Lourie R, Florin TH. ER stress and the unfolded protein response in intestinal inflammation. Am J Physiol Gastrointest Liver Physiol. 2010;298(6):G820–32. 10.1152/ajpgi.00063.2010 20338921

[60] Xu Y, Wang C, Li Z. A new strategy of promoting cisplatin chemotherapeutic efficiency by targeting endoplasmic reticulum stress. Mol Clin Oncol. 2014;2(1):3–7. 24649299 10.3892/mco.2013.202 PMC3916193

[61] Kawada M, Yamagoe S, Murakami Y, Suzuki K, Mizuno S, Uehara Y. Induction of p27Kip1 degradation and anchorage independence by Ras through the MAP kinase signaling pathway. Oncogene. 1997;15(6):629–37. 9264403 10.1038/sj.onc.1201228

[62] Lenferink AE, Busse D, Flanagan WM, Yakes FM, Arteaga CL. ErbB2/neu kinase modulates cellular p27(Kip1) and cyclin D1 through multiple signaling pathways. Cancer Res. 2001;61(17):6583–91. 11522658

[63] Wang X, Martindale JL, Holbrook NJ. Requirement for ERK activation in cisplatin-induced apoptosis. J Biol Chem. 2000;275(50):39435–43. 10993883 10.1074/jbc.M004583200

[64] van den Brink MR, Kapeller R, Pratt JC, Chang JH, Burakoff SJ. The extracellular signal-regulated kinase pathway is required for activation-induced cell death of T cells. J Biol Chem. 1999;274(16):11178–85. 10196203 10.1074/jbc.274.16.11178

[65] Cagnol S, Chambard JC. ERK and cell death: mechanisms of ERK-induced cell death—apoptosis, autophagy and senescence. FEBS J. 2010;277(1):2–21. 10.1111/j.1742-4658.2009.07366.x 19843174

