# Supplementary figures and images for: Flavokawain C Inhibits Cell Cycle and Promotes Apoptosis, Associated with Endoplasmic Reticulum Stress and Regulation of MAPKs and Akt Signaling Pathways in HCT 116 Human Colon Carcinoma Cells
Source: PLoS One. 2016 Feb 9;11(2):e0148775. doi: 10.1371/journal.pone.0148775 (PMC4747580; doi:10.1371/journal.pone.0148775)

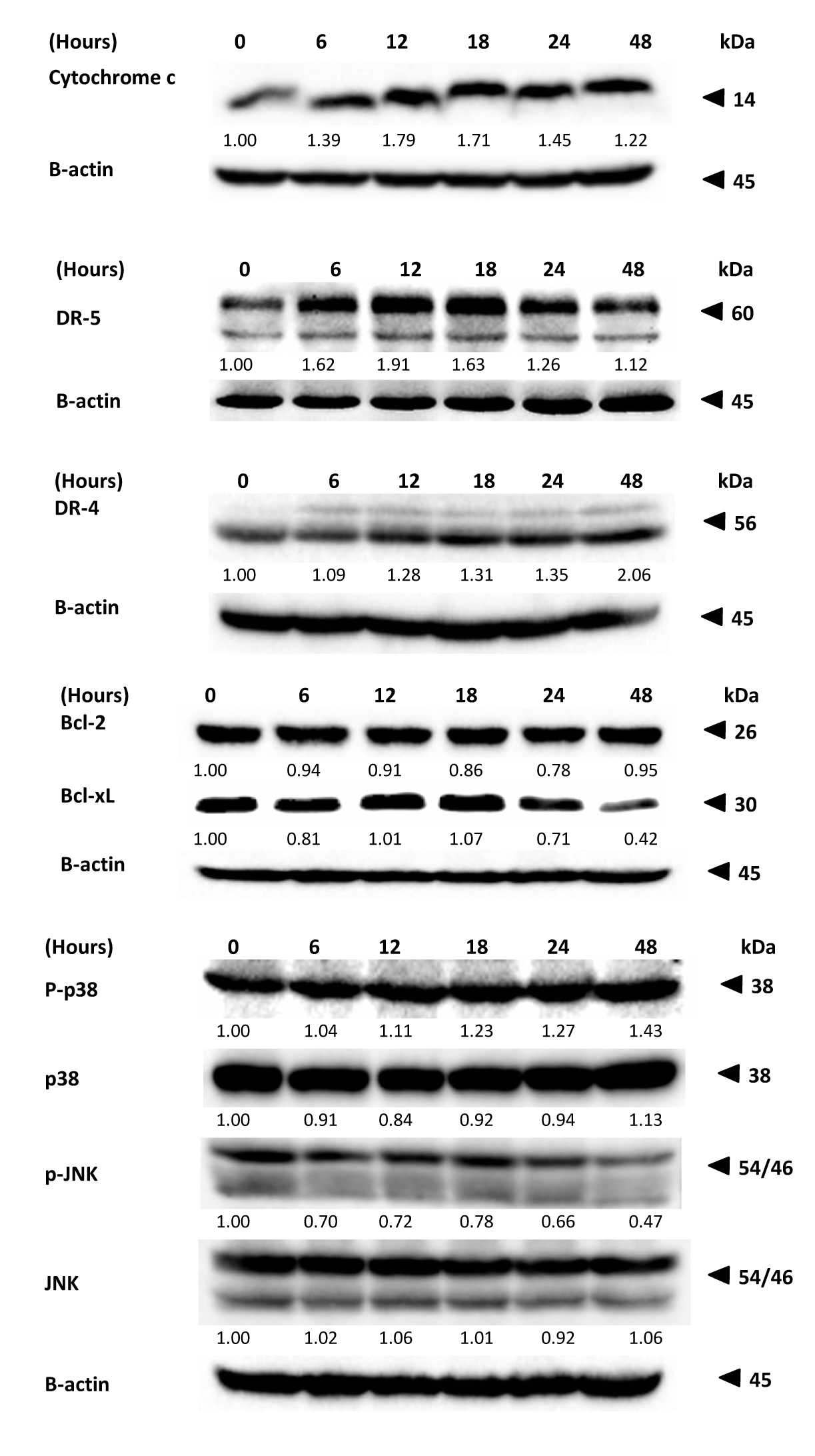

Supplement: S1 Fig — HCT 116 cells were treated with FKC (60μM) for indicated time points, and followed by protein extraction and western blot analysis. The results from representative experiments were expressed relative to the proteins level at 0hr after normalization to β-actin signals. Further validation will be needed to confirm the effects of FKC on these proteins. (TIF) [file pone.0148775.s001.tif]
